# Lineage-Specific Regulation of Epigenetic Modifier Genes in Human Liver and Brain

**DOI:** 10.1371/journal.pone.0102035

**Published:** 2014-07-23

**Authors:** Matthias K. Weng, Karthick Natarajan, Diana Scholz, Violeta N. Ivanova, Agapios Sachinidis, Jan G. Hengstler, Tanja Waldmann, Marcel Leist

**Affiliations:** 1 Doerenkamp-Zbinden Chair for *In Vitro* Toxicology and Biomedicine, University of Konstanz, Konstanz, Germany; 2 Center of Physiology and Pathophysiology, Institute of Neurophysiology, University of Cologne (UKK), Cologne, Germany; 3 Nycomed-Chair for Bioinformatics and Information Mining, Dept. of Computer and Information Science, University of Konstanz, Konstanz, Germany; 4 Leibniz Research Centre for Working Environment and Human Factors (IfADo), Technical University of Dortmund, Dortmund, Germany; University of Kansas Medical Center, United States of America

## Abstract

Despite an abundance of studies on chromatin states and dynamics, there is an astonishing dearth of information on the expression of genes responsible for regulating histone and DNA modifications. We used here a set of 156 defined epigenetic modifier genes (EMG) and profiled their expression pattern in cells of different lineages. As reference value, expression data from human embryonic stem cells (hESC) were used. Hepatocyte-like cells were generated from hESC, and their EMG expression was compared to primary human liver cells. In parallel, we generated postmitotic human neurons (Lu d6), and compared their relative EMG expression to human cortex (Ctx). Clustering analysis of all cell types showed that neuronal lineage samples grouped together (94 similarly regulated EMG), as did liver cells (61 similarly-regulated), while the two lineages were clearly distinct. The general classification was followed by detailed comparison of the major EMG groups; genes that were higher expressed in differentiated cells than in hESC included the acetyltransferase KAT2B and the methyltransferase SETD7. Neuro-specific EMGs were the histone deacetylases HDAC5 and HDAC7, and the arginine-methyltransferase PRMT8. Comparison of young (Lu d6) and more aged (Ctx) neuronal samples suggested a maturation-dependent switch in the expression of functionally homologous proteins. For instance, the ratio of the histone H3 K27 methyltransfereases, EZH1 to EZH2, was high in Ctx and low in Lu d6. The same was observed for the polycomb repressive complex 1 (PRC1) subunits CBX7 and CBX8. A large proportion of EMGs in differentiated cells was very differently expressed than in hESC, and absolute levels were significantly higher in neuronal samples than in hepatic cells. Thus, there seem to be distinct qualitative and quantitative differences in EMG expression between cell lineages.

## Introduction

Epigenetic modifier genes (EMG) encode the proteins that organize and maintain the chromatin structure of cells. They play a key role in the regulation of transcription and they ensure lineage fidelity by controlling the accessibility of DNA in the cell. During the early development of the zygote, genes that play a role in the maintenance of pluripotency are downregulated, whereas genes that are responsible for first cell fate decisions (germ layers) are upregulated. Other cell identifier genes are upregulated during the cellular maturation phase. Such waves of transcriptional changes are also found in differentiating embryonic stem cells (ESC) [1]. They are guided and controlled by chromatin structure, which regulates the accessibility of the underlying DNA to sequence-specific regulator proteins such as transcription factors (TFs) or the transcriptional initiation complex [2]. The two classical, simplified variants of chromatin are transcriptionally active “open” euchromatin that allows TF binding and silenced “closed” heterochromatin that prevents binding of TFs to the corresponding DNA sequences [3].

Chromatin structure is highly dynamic. The regulatory mechanisms include DNA methylation [4], post-translational modifications (PTM) of histones [5], chromatin remodeling [6], exchange of histone variants [7] and actions of non-histone structural proteins [8], [9]. They have an important impact on gene expression by affecting DNA accessibility. These control mechanisms, that are independent of the primary DNA sequence, are jointly termed “epigenetics” [10].

The nucleosome is the functional unit of chromatin and consists of DNA wrapped around an octameric histone core. The unstructured N-terminal tails of the histones protrude from this “core” and are targets of multiple post-translational modifications (PTM) [11]. Specific “writer”-enzymes are responsible for methylation, acetylation and ubiquitination of specific lysine and arginine residues or phosphorylation of serine and threonine residues [5]. In contrast, there are also enzymes that remove those PTM from the histone tails. These are termed “erasers”. Many different writers and erasers exist for one and the same modification [5]. For instance, at least 10 different enzymes methylate lysine 4 of histone H3 (H3K4me). The reason for this huge redundancy is still unclear, but lysine methyltransferases (KMTs) could have a cell type specific function.

Effector proteins that bind to a certain modification or a combination of modifications are called “readers”. They are able to translate the histone marks set by writers to higher order chromatin structures. Readers can range from structural proteins, like the heterochromatin protein-1 (HP-1) [12], high-mobility group (HMG) proteins [13] or DEK [8], to multi-subunit complexes that alter chromatin structure in an ATP-dependent manner (chromatin remodeling) [14]. Both, proteins that read histone PTMs and subunit composition of chromatin remodeling complexes display a high functional redundancy. A well described example of tissue specific subunit assembly is the SWI/SNF-complex [15]. Depending on the cellular lineage and the developmental stage the SWI/SNF-complex is composed of varying types of subunits [16]. Another study also revealed a high degree of diversity in the peripheral subunits of KMT complexes [17]. Since many cellular processes impinge on and depend upon chromatin structure, there is no universally agreed list of all EMGs. An incomplete, but representative set of major EMGs has been defined for purposes of expression fingerprinting [18]. This list has been used to follow drastic expression changes during the course of human neurodevelopment [18]. This study suggested that there may be some mature neuron-specific EMGs.

The present study is built on these earlier findings and uses the panel of EMGs to compare their expression in neuronal cells to that of an entirely different cell lineage. We complemented existing data on cortex (Ctx) samples with those derived from pure and homogeneous human cultured neurons and contrasted those to a primary and a hESC-derived hepatocyte culture. All samples expressed typical cell type specific marker genes on mRNA as well as on protein level. Immunofluorescence stainings also showed that the cell lines that were differentiated *in vitro* are highly homogeneous and therefore well suited for this kind of approach. This allowed us to investigate the pattern of EMG expression, and compare it to hESC as reference population. Detailed data are provided on multiple groups of EMGs, and our unbiased approach demonstrates a high degree of cell type specificity of expression profiles.

## Materials and Methods

### Cultivation and differentiation of LUHMES cells

Lund human mesencephalic cells (LUHMES) are a subclone of the human mesencephalic-derived cell line MESC2.10, characterized at and originating from Lund University (Lund, Sweden) [19]. They are conditionally immortalized with a v-myc retroviral vector. Tetracycline is used to shut down v-myc expression and trigger differentiation into dopaminergic neurons. Cells were cultured exactly as described earlier [20], [21]. Nunclon (Nunc, Roskilde, Denmark) plastic cell culture flasks and multi-well plates – pre-coated with 50 µg/ml poly-L-ornithine and 1 µg/ml fibronectin (Sigma-Aldrich, St. Louis, MO, USA) in water for 3 h – were used. After removal of the coating solution, culture flasks were washed once with water and air-dried before seeding the cells. Proliferation medium consisted of Advanced Dulbecco’s modified Eagle’s medium/F12, 1x N-2 supplement (Invitrogen, Karlsruhe, Germany), 2 mM L-glutamine (Gibco, Rockville, MD, USA) and 40 ng/ml recombinant basic fibroblast growth factor (R&D Systems, Minneapolis, MN, USA).

Cells were passaged 1∶10 when they reached 80% confluency. For differentiation, 8×10^6^ cells were seeded into a T175 flask in proliferation medium and differentiation was started after 24 h, (d0), by changing to differentiation medium. Differentiation medium, consisted of Advanced Dulbecco’s modified Eagle’s medium/F12, 1x N-2 supplement, 2 mM L-glutamine, 1 mM dibutyryl-cAMP (Sigma-Aldrich), 1 µg/ml tetracycline (Sigma-Aldrich) and 2 ng/ml recombinant human GDNF (R&D Systems). After 2 days of cultivation in culture flasks, cells were trypsinized (0.05% trypsin-EDTA; GIBCO, Rockville, MD, USA) and seeded into pre-coated multi-well plates at a cell density of 1.5×10^5^ cells/cm^2^, if not otherwise indicated. Fresh differentiation medium was added every second day.

### Fresh human hepatocyte samples

Primary human hepatocytes were isolated from resected human liver tissue by EGTA/collagenase perfusion according to a published standard operation procedure [22]. All donors gave written consent and the project has been approved by the ethics committee of the faculty of medicine at the technical university of Munich (TUM00253/09). The use of the material for the present study was also approved by the institutional review board (IRB) of the University of Konstanz (statement IRB78/12). Hepatocyte culture and immunostaining were performed as described [23], [24]. Briefly, cells were cultured on glass dishes in 6-well plates between two layers of collagen soft gel, fixed in 4% paraformaldehyde for 20 minutes and washed three times in PBS pH 7.4. Permeabilisation was performed with 0.3% Triton X-100 in PBS for 15 minutes. After three washing steps in PBS, the cells were blocked with 3% BSA in PBS for 1 h. Subsequently, the cells were incubated overnight at 4°C with goat anti-human DPPIV (dipeptidyl peptidase-4; R&D system; cat. no. AF1180; Nordenstadt – Germany; diluted 1∶100) to stain for bile canaliculi, or rabbit anti-human serum albumin (Abcam; cat. no. ab2406; Cambridge – UK; diluted 1∶200; [25]) to stain hepatocytes. The secondary antibodies were donkey anti-goat Alexa Fluor 488 (Dianova, cat. no. 705–546–147; Hamburg – Germany; diluted 1∶200) and donkey anti-rabbit Cy3 (Dianova, cat. no. 711–166–152; Hamburg – Germany; diluted 1∶250). Images were recorded on a confocal microscope (FV-1000, Olympus; Hamburg, Germany) equipped with a 40x lens.

### Differentiation of H9 cells to hepatocyte-like islets

Hepatocyte-like cells were obtained from H9 human embryonic stem cells using the differentiation protocol of Sullivan et al. [26] with the difference that the second differentiation step with 1% DMSO was performed for eight days instead of seven days. Briefly, a first differentiation step (3 days) was performed in RPMI medium with 1x B27, Wnt3a (50 ng/ml) and activin A (100 ng/ml) to generate definitive endoderm. A second differentiation step (8 days) was done in Knockout-DMEM, 20% Knockout serum replacement (KOSR) and 1% DMSO to differentiate the definitive endoderm to hepatoblast-like cells. In a third differentiation step (7 days) hepatocyte-like cells were obtained by further cultivation in L-15, Hepatocyte growth factor (HGF;10 ng/ml) and Oncostatin M (20 ng/ml).

### Human brain samples

The cortex of three neurologically healthy control individuals (mean age 75±10 years), provided by the German Brain-Net (Munich, Germany) was used for analysis. As described earlier [18] post-mortem cortex samples had been obtained after written consent of the subjects and the next of kin, in adherence to the guidelines laid down in the Declaration of Helsinki on human research ethics. The use of the material was approved by the institutional review board (IRB) of the University of Konstanz (statement IRB78/12). RNA was extracted from frozen tissue and converted to cDNA as described.

### Immunostaining of neural cells

Cells were grown and differentiated on glass cover slips and fixed with PBS, 4% para-formaldehyde, 2% sucrose for 15 minutes. After permeabilization with 0.2% Triton-X-100 in PBS for 7 minutes, the cells were blocked for one hour in blocking solution (PBS, 1% BSA, 0.1% Triton-X-100). Primary and secondary antibodies (see Table S1) were diluted in blocking solution and incubated for one hour each. DNA was stained with Hoechst-33342 and mounted with Fluorsave reagent (Calbiochem).

Images were taken with an IX81 inverted microscope (Olympus, Hamburg, Germany) equipped with a 40x air objective and processed using Cell^P^ imaging software (Olympus). For confocal microscopy, cover slips were mounted using Vectashield (containing DAPI), images were taken with a Zeiss LSM 510Meta confocal microscope equipped with a Plan Apochromat 63x, NA 1.4 oil DIC lens. Images were processed, using Adobe Photoshop CS2, and antigens are displayed in false colors as indicated by the antigen label in the figures.

### Reverse transcription and quantitative RT-PCR

In a previous study, PCR analysis has been shown to have a much higher sensitivity and robustness than data extraction from microarray-based gene expression studies [18]. Therefore PCR was used here as method of choice. For reverse transcription quantitative PCR (qPCR) analysis, RNA was extracted with the RNeasy mini Kit (Qiagen, Hilden, Germany). The cDNA synthesis was performed using the cDNA synthesis kit from SABiosciences or from Invitrogen. Primersequences (see Table S1) were either picked from a primer database (http://primerdepot.nci.nih.gov) or designed according to the following requirements: exon-spanning primers were designed manually and optimized for melting temperature and primer dimerization by using the Primer3 tool (http://primer3.sourceforge.net/). Afterwards they were tested for nonspecific amplification products through melt curve analysis and agarose gel electrophoresis. All qPCRs were run in a CFX96 thermal cycler (Biorad, München, Germany) using the following settings: 1x (30 sec 98°C), 40x (2 sec 98°C, 5 sec 60°C) or the settings described for the RT^2^Profiler PCR arrays by SABiosciences. A large part of the primer sets used here is available on pre-assembled plates as RT^2^Profiler PCR Arrays (“Human Neurogenesis and Neural Stem Cell” (PAHS-404A), “Human Epigenetic Chromatin Modification Enzymes” (PAHS-085A), and “Human Epigenetic Chromatin Remodeling Factors” (PAHS-086A), all from SABiosciences, Frederick, MD, USA). Data in figures are shown as means ± SEM of three independent differentiations. For statistical analysis, we used the data calculated with the ΔCt method and performed two-tailed t-test with Welch correction for different variances between hESC, LUHMES, Hep-like, huHep or Ctx. In a second step we corrected the p-values for multiplicity via Benjamini-Hochberg FDR (false discovery rate)-correction. Detailed information on fold regulation values and statistical analysis of all EMGs can be found in figures S1 and S2.

### Normalization of qPCR data for cell type comparisons

The threshold cycle values (Ct) determined with the CFX96 optical system software (Bio-Rad) were exported to Microsoft Excel for further analysis. To evaluate the stability of the 5 reference genes present on the array, the *geNorm-*macro for Microsoft Excel was used [27]. Gene expression stability (M) was calculated with *geNorm*, and the genes were ranked from best to worst, based on the M value. *GeNorm* determines the individual stability of a gene within a pool of genes, and calculates the stability according to the similarity of their expression profile by pair-wise comparison, using the geometric mean as a normalizing factor. The gene with the highest M, i.e. the least stable gene, is then excluded in a stepwise fashion until the most stable genes are determined. This way we ended up with three reference genes (HPRT1/RPL13A/GAPDH) that showed M-values ranging from 0.41 to 0.59 depending on the data set analyzed. Calculation of the relative expression values (fold change or (2^−(ΔΔCt)^)) of all genes was performed using the comparative Ct method [28], [29].

### Bioinformatics and data analysis

For the visualization of qPCR data, generated with the ΔΔCt method, we implemented a heat map solution as graphical representation. To express gene regulation, we used 256 steps for blue (down-regulation) and red (up-regulation). The scaling was adapted so that a manually chosen threshold value in each group (e.g. 20-fold up-regulation) defined the maximum color saturation. Then, color scaling steps were linearly mapped to gene regulation values between 1 and the threshold value in red and below 1 in blue. Genes regulated not significantly after FDR-correction were marked with “n.a.” for not regulated. The hierarchical clustering analysis based on our 156 histone modifier genes was performed as previously described [30]. Average linkage was used as agglomeration rule for the clustering analysis. Euclidean measure was used to calculate distance for transcripts (rows of the heat map) and samples (columns of the heat map). The gene expression level was indicated by yellow for low expression and red for high expression.

### GEO2R analysis

In order to compare the data we collected with previous gene-array studies on dopaminergic neurons we used the Gene Expression Omnibus (GEO) platform of NCBI (http://www.ncbi.nlm.nih.gov/geo/). GEO is a public genomic data repository that also provides tools to generate expression profiles of existing array data sets for comparison. For this purpose we picked microarray data (GSE51214) from a recently published work that describes the generation of dopaminergic neurons from human induced pluripotent stem cells (iPSC) [31]. We chose this data set not only because it contains human samples with a dopaminergic phenotype but also because it includes iPSC- and human fetal mesencephalon (huMES) samples. This way we were able to normalize differentiated cells (DA d42) and fetal tissue samples from the mesencephalon (huMES) to pluripotent stem cells. This made both of them comparable to our hESC-normalized samples. For the analysis we used the GEO2R tool and followed the instructions provided on the website. The results of our analysis were downloaded and the expression values for our 156 HMGs were extracted for comparison (5 of them could not be found in the data and were excluded). Lu d6 and Ctx expression values were calculated as fold-changes and then log2-transformed in order to make them comparable to the log2 fold-change values of the microarray data. Finally, we generated a heat map from these four sample sets (Fig. S5). The heat map depicts every log fold change <1 with N for “not regulated”, as we defined a cutoff of 2-fold expression changes (1 on the log2 scale).

## Results and Discussion

### Distinct profiles of epigenetic modifier expression in liver and brain

**Figure 1:**
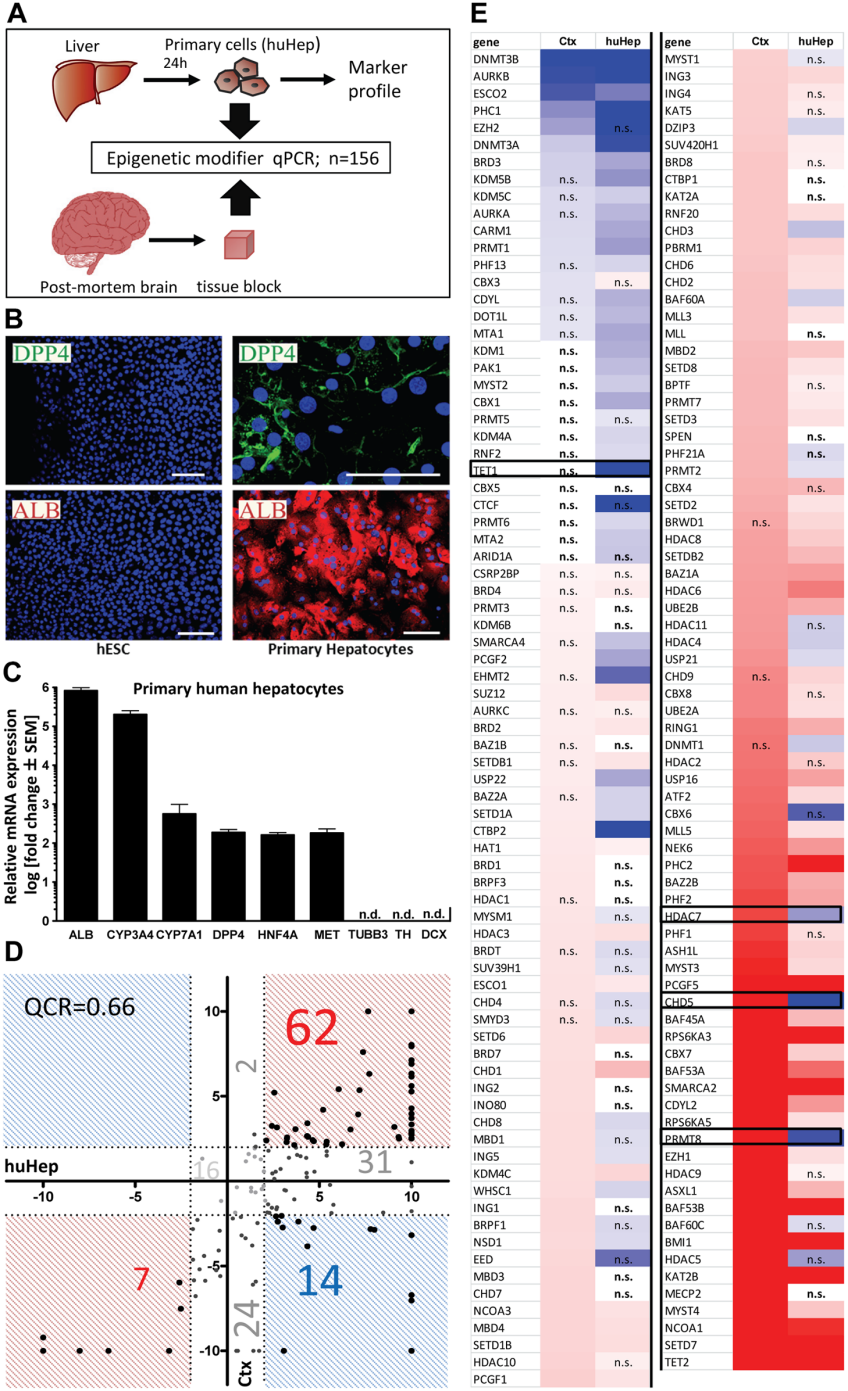
Comparison of epigenetic modifier transcript levels between liver and brain. (**A**) Schematic diagram showing sample preparation and analysis of a set of 156 epigenetic modifier genes. (**B**) Human hepatocytes were stained 24 h after plating with antibodies specific for dipeptidyl peptidase (DPP4) or albumin (ALB). Nuclei were stained with the DNA dye H-33342 (blue). Data are representative for preparations from three different donors. Scale bars: 100 µm. (**C**) The mRNA was isolated from three preparations of freshly-isolated hepatocytes and analyzed by RT-qPCR for hepatic (ALB, CYP3A, CYP7A1, DPP4, HNF4, MET) and neuronal (TH, DCX, TUBB3) differentiation markers. Gene expression levels are indicated relative to hESC as reference cell line and a set of three reference genes (HPRT, RPL13A, GAPDH) was used for internal calibration. (**D**) Transcript levels of epigenetic modifiers were measured by RT-qPCR in human cortex (Ctx), liver (huHep) and embryonic stem cells (hESC). Data for Ctx and huHep are indicated as relative change compared to hESC (as reference cell). For comparative display, a scatter plot was constructed so that differentially expressed genes that show pos. association (between Ctx and huHep) are found in red fields, and those that differed in the sense of regulation fall into blue fields. Values of>10 were set to 10. For quadrant count ratio analysis (QCR) only expression values >2 or <−2 were included. (**E**) The data measured in D were plotted as heat map, sorted according to rel. Ctx expression levels. Transcripts that were >2-fold higher expressed in tissue than in hESC are marked in red, >2-fold lower expression is marked in blue. The color scale ranges from a fold regulation of −20 (dark blue) to +20 (dark red). Measures of variance and p-values are indicated in the supplemental material, genes not regulated significantly (vs. hESC) are displayed as “n.s.”. Specific examples of differential regulation between Ctx and huHep are emphazised by black boxes.

Recently, we determined the expression profiles of epigenetic modifier genes (EMG) in one type of tissue (neuronal) for different developmental stages, e.g. mature human cortex (Ctx) versus immature neural precursors [18]. To investigate potential tissue specificity, we compared now primary human hepatocytes (huHep) to human Ctx samples (Fig. 1A). For a better comparison of the expression levels across different lineages, all data were normalized to the expression levels of hESC as common fixed reference point. Thus, data are given as fold change difference compared to hESC. To allow other comparisons and a free choice of reference cell, we also provide absolute expression data for all cells and for the hESC (Fig. S3). First we confirmed the differentiation state of the hepatocyte samples used in this study. Immunofluorescence staining of huHep showed a ubiquitous expression of the two hepatic markers albumin (ALB) and dipeptidyl-peptidase 4 (DPP4) (Fig. 1B). Gene expression levels of these two proteins and of further hepatic lineage markers (CYP3A, CYP7A1, HNF4, MET) were also high compared to hESC, which were used here as neutral reference cell type. In contrast to this, transcripts of neuronal genes (TH, DCX, TUBB3) were not detectable (Fig. 1C). After having confirmed the overall phenotype of the liver samples, we measured the expression levels of 156 epigenetic modifier genes by RT-qPCR. These were then compared to Ctx samples that have been characterized earlier [18]. A scatter plot analysis showed that 14 genes (9%) were regulated in opposite directions (Fig. 1D). These transcripts were all downregulated in huHep and upregulated in Ctx. Altogether, 69 genes (45%) were regulated in the same direction. For individual comparison of EMGs, the transcript levels were displayed as a heat map (Fig. 1E). For instance, the mRNA levels of the protein arginine methyltransferase PRMT8 and for the chromodomain/helicase/DNA-binding domain protein CHD5 were both highly expressed in Ctx but very low in huHeps (Fig. 1E). Both proteins have indeed been reported to be specific for neuronal tissue in mice [32], [33]. Moreover, we found recently that both genes are not yet upregulated in neural progenitor cells [18]. Other examples for genes with clearly higher expression levels in Ctx than in huHep were HDAC7, a histone deacetylase which is reported to have a neuroprotective function [34] and TET1, a methylcytosine dioxygenase that initializes DNA demethylation and is supporting neuronal activity-regulated genes [35]. Because TET1 was already highly expressed in hESC (Ct = 23) there was no significant upregulation visible compared to Ctx. Both genes are expressed in both tissues as well as in hESC (Fig. S3). In summary, the genes with reported specific neuronal function (PRMT8, CHD5, HDAC7 and TET1) were only expressed (CHD5 and PRMT8) or much higher expressed (HDAC7 and TET1) in Ctx. In contrast, genes with known ubiquitous functions during development were also upregulated in huHep samples and showed a low expression level only in hESC (e.g. KAT2B, PHC2, SETD7). This suggests that EMG transcription profiles may discriminate between different cell lineages. Notably, individual EMGs are unlikely to be good lineage markers because each of them has functions required in multiple cell types to maintain and modify the structure of chromatin. However, for certain cellular functions a specific chromatin environment might be necessary that can only be established by specific EMGs enriched in the respective tissues. Therefore, much like transcription factors and other cellular components that help to define cellular lineage, there may be sets of EMGs that are associated with certain cell types or cellular developmental stages. These sets of genes might be called cell type specific chromatin modifier fingerprints.

### Comparison of the EMG profile of two hepatocyte populations

**Figure 2:**
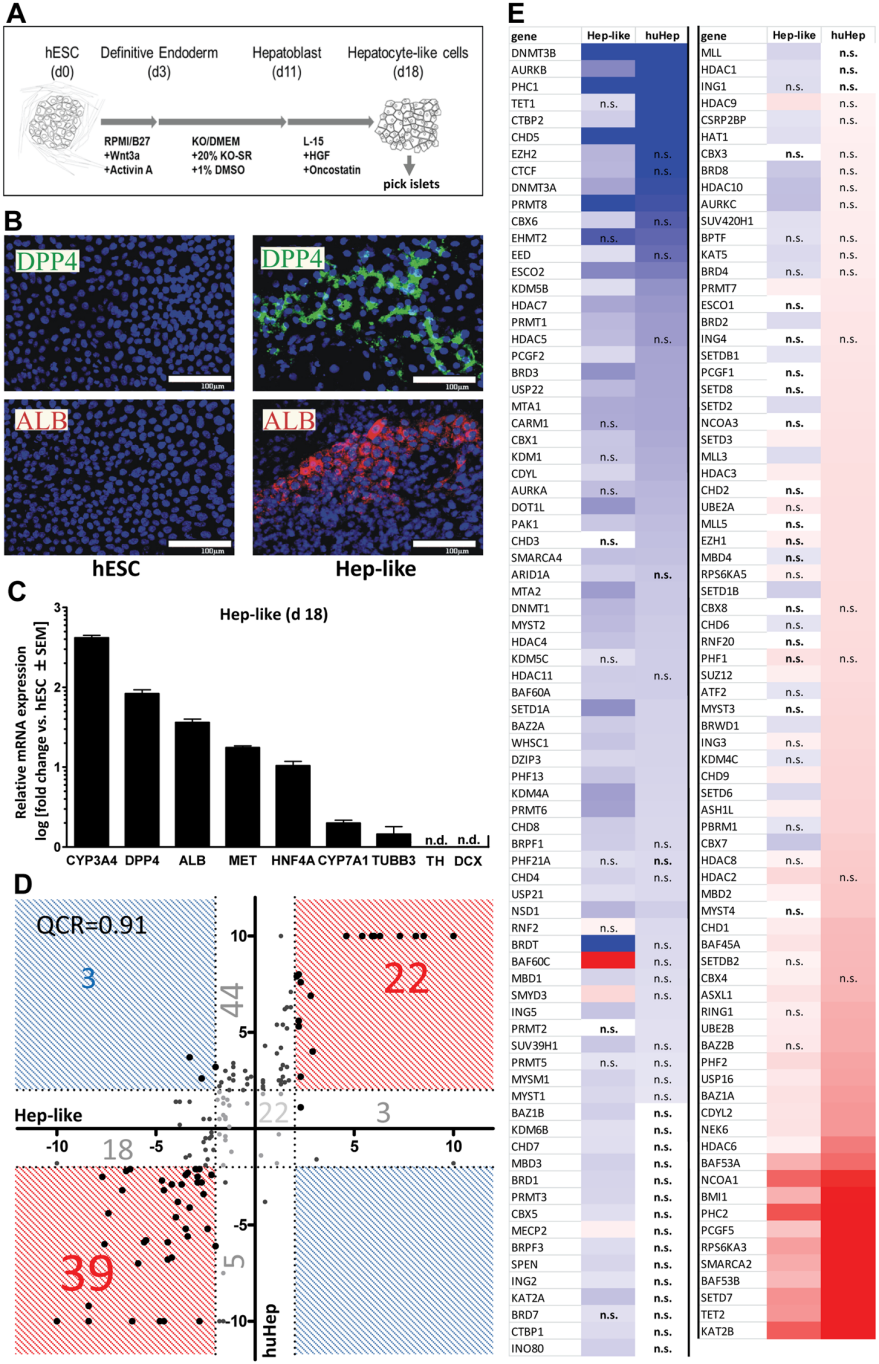
Comparison of histone modifier sets between two hepatic cell cultures. (**A**) Differentiation scheme of hESC towards Hepatocyte-like cells (Hep-like). (**B**) Hep-like cells were generated from hESC and stained with antibodies specific for DPP4 and albumin. Nuclei were stained with the DNA dye H-33342 (blue). Scale bars: 100 µm. (**C**) RT-qPCR data from three independent experiments for hepatic (ALB, CYP3A, CYP7A1, DPP4, HNF4, MET) and neuronal (TH, DCX, TUBB3) markers. Relative gene expression was calculated using hESC as a calibrator and a set of three reference genes (HPRT, RPL13A, GAPDH). (**D**) Transcript levels of epigenetic modifiers were measured by RT-qPCR in human liver (huHep), Hep-like islets (Hep-like) and embryonic stem cells (hESC). Data for huHep and Hep-like are indicated as relative change compared to hESC (as reference cell). For comparative display, a scatter plot was constructed so that differentially expressed genes that show pos. association (between Hep-like and huHep) are found in red fields, and those that differed in the sense of regulation fall into blue fields. Values of >10 were set to 10. For quadrant count ratio analysis (QCR) only expression values >2 or <−2 were included. (**E**) The data measured in D were plotted as heat map, sorted according to rel. huHep expression levels. Transcripts that were >2-fold higher expressed in tissue than in hESC are marked in red, >2-fold lower expression is marked in blue. The color scale ranges from a fold regulation of −20 (dark blue) to +20 (dark red). Measures of variance and p-values are indicated in the supplemental material, genes not regulated significantly (vs. hESC) are displayed as “n.s.”.

If the profile of EMG expression is lineage-specific, then the differences between huHep and Ctx should be larger than between huHep and an independently-derived, but phenotypically related cell culture [26]. To this end, we compared huHep and a stem cell-derived culture of hepatocyte-like cells (Hep-like). The latter was differentiated from human embryonic stem cells (hESC) (Fig. 2A) [26]. Similar to huHep, the Hep-like cells strongly stained for hepatic markers like albumin and DPP4 (Fig. 2B). The staining was distributed in island-like patches among less differentiated cells. These islets were picked for further analysis. RT-qPCR showed that hepatic markers were upregulated (Fig. 2C). Neuronal markers were either not detectable or expressed at low levels (Fig. 2C). The entire set of EMGs was then measured, and scatter plot analysis showed that the two hepatic cell preparations had a very similar expression profile (Fig. 2D). The quadrant count ration (QCR) of 0.91 (compared to 0.66 between Ctx and huHep) provided strong evidence for a highly related machinery of epigenetic regulation. Out of 156 genes only three were regulated in opposite directions, while 61 were regulated in the same direction (Fig. 2D). The discrepant genes (CBX7, SETD1B, SETD6) were downregulated in Hep-like cells and upregulated in mature huHeps. However, the absolute expression levels (Fig. S3) revealed that SETD1B and SETD6 are expressed in both hepatic cell types. In contrast, CBX 7 is weakly expressed in the differentiated Hep-like cells compared to huHeps. CBX7 is a subunit of the polycomb repressive complex 1 (PRC1) and an epigenetic reader protein of the histone 3 lysine 27 trimethylation (H3K27me3) [36]. SETD1B specifically methylates lysine 4 at histone 3 (H3K4me) [37] and SETD6 mono-methylates lysine 7 of H2AZ (H2AZK7me1) and is important for mESC maintenance as well as lineage commitment during differentiation [38]. Another interesting observation was the stronger upregulation of BAF60C in the more immature Hep-like islet cells. BAF60C is a subunit of the conserved SWI/SNF-complex whose composition is highly dependent on the cellular and developmental context [15]. The specific upregulation in hepatic precursor cells indicates a developmental stage-specific function of BAF60C. Notably, PRMT8, HDAC7 and CHD5 that were earlier found to be specifically upregulated in Ctx tissue were downregulated in Hep-like as well as in huHep cells. This indicates that these genes may have indeed a more prominent role in neuronal tissue.

### Similarities of EMG regulation in different neuronal cells

**Figure 3:**
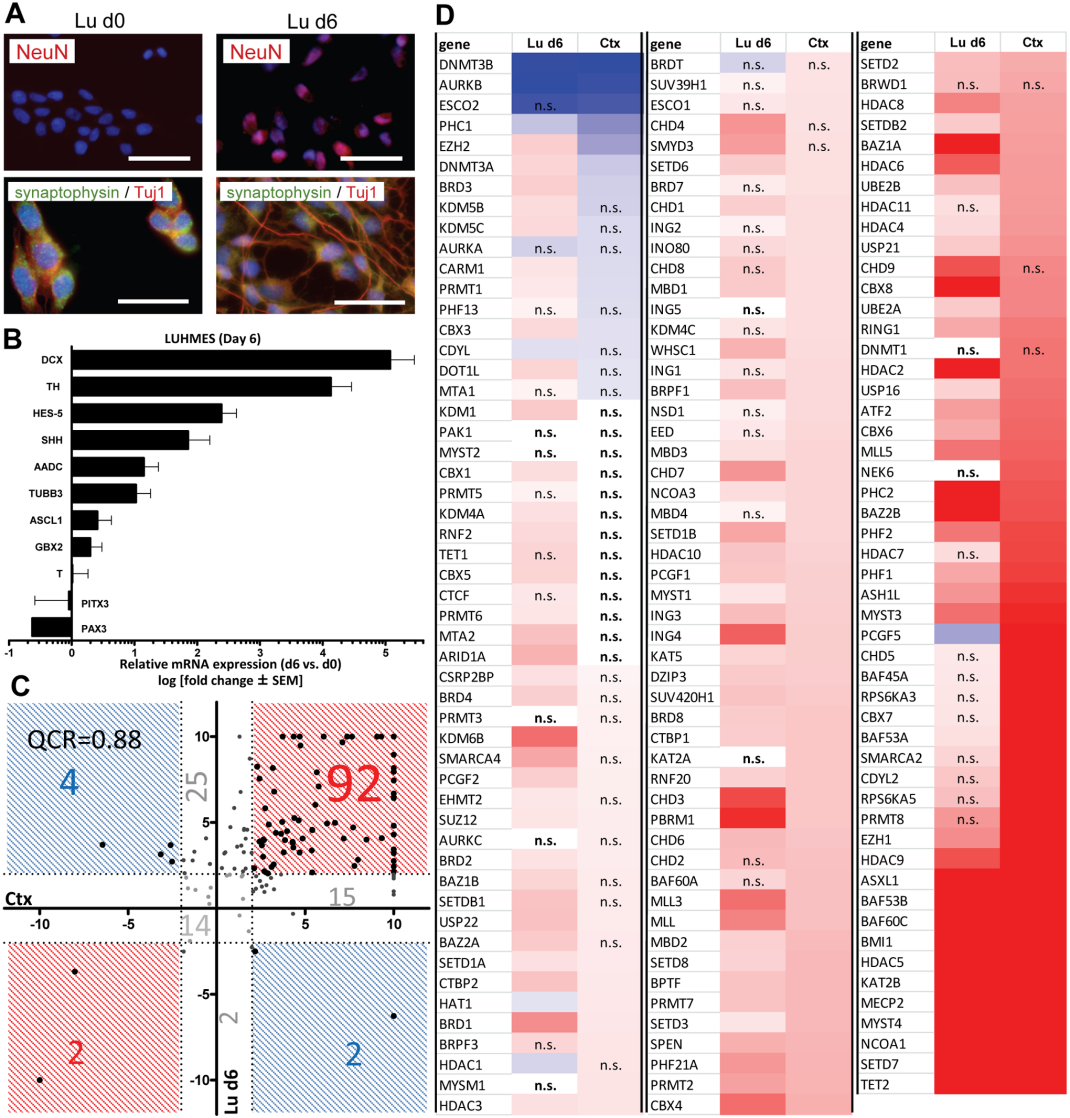
Comparison of histone modifier sets between LUHMES cells and Cortex samples. (**A**) LUHMES cells (Lu0) were differentiated for 6 days (Lu d6) and stained with antibodies specific for NeuN, synaptophysin and TUJ1. Nuclei were stained with the DNA dye H-33342 (blue). Scale bars: 100 µm. (**B**) RT-qPCR data from three independent experiments for dopaminergic and neuronal differentiation markers. Relative gene expression was calculated using day0 as a calibrator and a set of three reference genes (HPRT, RPL13A, GAPDH) (**D**) Transcript levels of epigenetic modifiers were measured by RT-qPCR in human Cortex (Ctx), LUHMES day6 (Lu d6) and embryonic stem cells (hESC). Data for Ctx and Lu d6 are indicated as relative change compared to hESC (as reference cell). For comparative display, a scatter plot was constructed so that differentially expressed genes that show pos. association (between Ctx and Lu d6) are found in red fields, and those that differed in the sense of regulation fall into blue fields. Values of >10 were set to 10. For quadrant count ratio analysis (QCR) only expression values >2 or <−2 were included. (**E**) The data measured in D were plotted as heat map, sorted according to rel. Ctx expression levels. Transcripts that were >2-fold higher expressed in tissue than in hESC are marked in red, >2-fold lower expression is marked in blue. The color scale ranges from a fold regulation of −20 (dark blue) to +20 (dark red). Measures of variance and p-values are indicated in the supplemental material, genes not regulated significantly (vs. hESC) are displayed as “n.s.”.

We investigated the EMG pattern of *in vitro* differentiated human neurons. This was of special interest, as this approach provided data on a pure and homogeneous neuronal human cell population, and we were curious whether this would confirm the gene expression pattern found in Ctx tissue samples. For this analysis, we chose LUHMES cells, a neuronal precursor cell line that can be differentiated into mature and functional dopaminergic neurons within 6 days [21], [39], [40], [41]. The differentiated cells (Lu d6) displayed specific neuronal markers like NeuN (FOXD3) and synaptophysin. The neuron-specific class III beta-tubulin (TUJ1) was strongly expressed, and stained the intricate neurite network of the day 6 cell population (Fig. 3A). The neuronal lineage identity was also confirmed by expression analysis of specific marker genes. Neuronal genes and controls of neurodifferentiation were upregulated while the early progenitor marker PAX3 was downregulated in postmitotic Lu d6 cells compared to proliferating precursor cells (Lu d0) at the start of differentiation (Fig. 3B). Lu d6 cells and Ctx showed a high concordance (QCR = 0.88) in EMG regulation. As many as 94 genes were equally regulated. Only 6 genes were regulated in opposite directions (Fig. 3C). Differentially regulated genes between the two cell types included the PcG ring finger protein 5 (PCGF5) or the bromodomain protein BRD3, the latter was upregulated in Lu d6 and downregulated in Ctx. However, the absolute expression levels did not differ much compared to PCGF5 (Fig. S3). BRD3 binds to hyperacetylated chromatin and facilitates RNA polymerase II (Pol II)-associated transcription [42] (Fig. 3D). PCGF5 is a component of the multimeric PcG complex that is involved in stable gene silencing [43]. A putative functional SNP in PCGF5 has recently been associated with Alzheimer’s disease [44]. Interestingly, PCGF5 was highly expressed in Ctx and downregulated in Lu d6. As already hypothesized for the hepatic cells, such differences might be due to the different maturation stages of the compared cells. From a cell culture perspective, the “young” Lu d6 are fully differentiated but may be less mature than the “old” adult brain tissue. These differences might be the result of an adaption of the neurons to environmental influences during life. Recent studies indeed showed an age-related reorganization in the human neuronal epigenome and neurons derived from human induced pluripotent stem cells require more time to reach a functionally mature state compared to rat neurons [45], [46]; it is also possible that the observed differences are due to the higher homogeneity of Lu d6 which represent only one cell type of the brain, while Ctx tissue contains multiple cell types. Another explanation for these differences might be that the LUHMES cells, although they show all properties of mature functional neurons, are derived from an immortalized neuronal precursor cell line which not fully represents the *in vivo* state compared to Ctx tissue. For this later reason, we did not focus here on comparisons of Lu d0 (transgene-expressing) and d6 cells. However, a complete set of data on the change of EMG during LUHMES differentiation is displayed in Fig. S4.

In order to compare our results on EMG expression in Lu d6 and Ctx with dopaminergic neurons from other sources we searched for published gene-array studies. We extracted data from a study on dopaminergic progenitors that were derived from induced pluripotent stem cells (iPSC) [31]. Additionally, we compared our data with fetal mesencephalic tissue from the same study and created a heat map with all four sample types (Fig. S5) More than 50% of the genes that were expressed with more than 2-fold differences were regulated in the same direction. We saw similar regulation patterns for the highest and lowest regulated genes in all four cell types (BAF53b, KAT2B, BMI1, DNMT3b and AURKB). Interestingly, the maturation-dependent switch (observed earlier in Ctx) from EZH2 to EZH1 was now also observed in iPSC derived dopaminergic neurons. This finding suggests that these cells are more mature than differentiated LUHMES cells (no EZH switch). Indeed the iPSC-derived neurons had been differentiated for 42 days instead of only 6 days of differentiation in LUHMES cells. In summary, our data show that the EMG expression patterns in adult Ctx are closely related to the one of a pure neuronal culture generated from neural precursor cells (LUHMES d6). Moreover, the data also shows similarities with gene array data of dopaminergic neurons. This confirmed our assumption that expression of EMG is likely to have a strong lineage-specific component.

### Cluster analysis of EMG expression across different cell types

**Figure 4:**
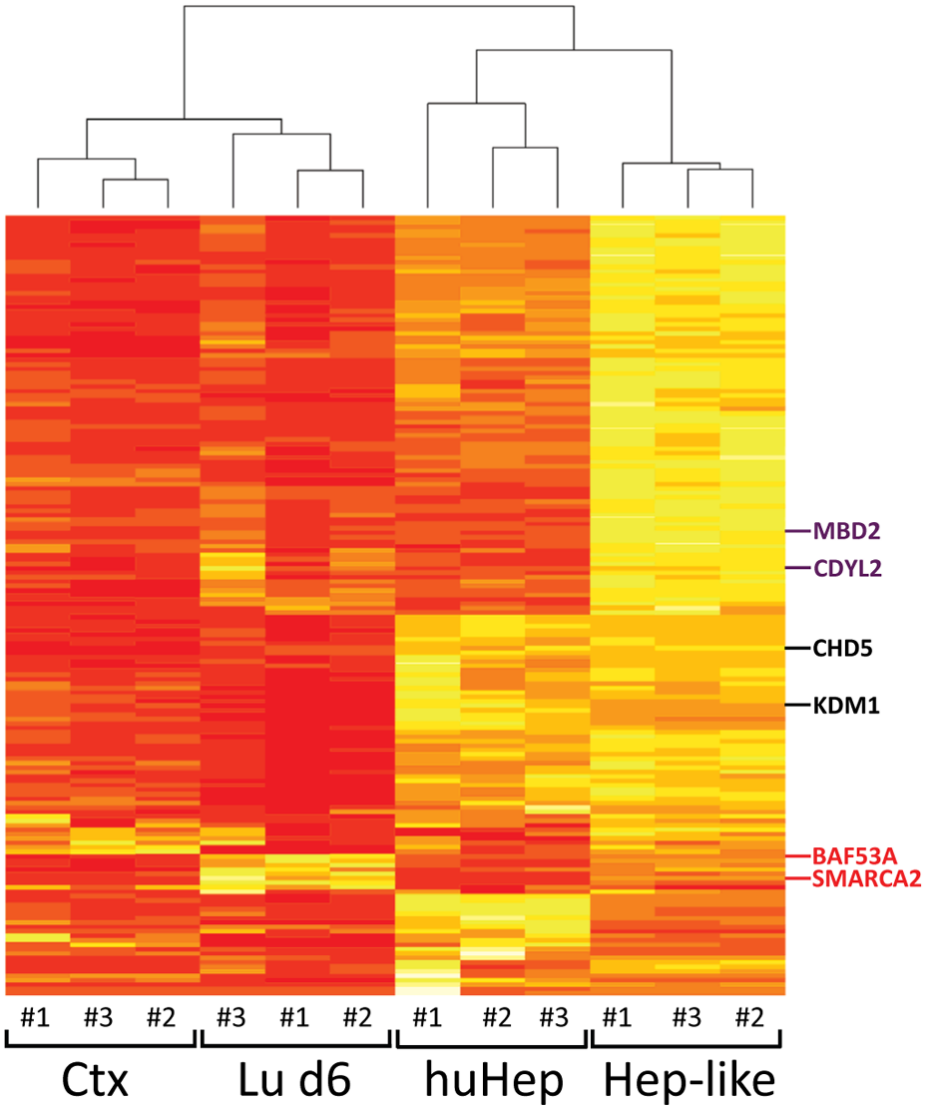
Comparison of epigenetic modifier gene expression across cell lineages. (**A**) The expression of 156 modifier genes was measured for 4 differentiated cell types as specified in Fig. 1–3, and transcript levels were all normalized to those of hESC. Three independent assays (#1–#3) were performed for each cell type, and the 12 data sets were represented as heat map. Red color indicates z-scores >0 and yellow color indicates z-scores <0. The data sets were clustered according to their Euclidean distances, as indicated by the dendrogram on top. The individual example genes depicted on the right in black show large differences between liver (huHep, Hep-like) and brain (Ctx, Lu d6). Genes with similarities in non-dividing cells (Ctx, Lu d6, huHep) are shown in purple; those with differences between primary and stem cell-derived cells are shown in red.

To further explore the relationships of EMG expression patterns between the cell types we used a more unbiased statistical method. Direct comparisons of expression levels were possible, as they had all been normalized to the same reference population, i.e. hESC. We used three datasets for each of the four cell types and determined Euclidean distances between all of them (Fig. 4). The cluster analysis showed that the individual data sets cluster according to their respective cell type. This gave evidence of the robustness of our analytical method, cell differentiation and sample preparation. In addition, this analysis revealed that Ctx and Lu d6 are indeed more related to each other than to huHep or Hep-like cells. The latter two also clustered together showing a clear separation of the hepatic from the neuronal cell types (Fig. 4). This method confirmed CHD5 [32], [33] to be regulated in a cell lineage-specific fashion. An example gene showing a similar segregation is the lysine specific demethylase KDM1 (also called LSD1). It targets mono- and dimethylated H3K4 and H3K9 [47], [48]. However, it needs to be noted that KDM1 is also expressed in some non-neuronal cells, as it is for example involved in terminal maturation of blood cells [49]. We also identified a big group of genes that were similarly expressed in Ctx, Lu d6, and huHep, but not in the Hep-like cells which are the most immature of the cell populations and had not yet reached the postmitotic stage. Two examples of such genes are the H3K9me-binder CDYL2 [50], [51] and MBD2, a binder of methylated DNA [52], [53]. Lastly, there were also a few genes whose expression levels differed mainly between less mature (Lu d6; Hep-like) and mature populations generated *in vitro* (Ctx, huHep) cells aged for decades in the human body. Two subunits (BAF45A/53A) of the SWI/SNF remodeling complex and one ATPase of the same complex (SMARCA2) were highly expressed in huHep and CTX and had low levels of expression in the other cells. SWI/SNF is one of the best described ATP-dependent chromatin-remodeling complexes and has many different targets and functions depending on the combinatorial assembly of its subunits [15]. Our data corroborated the known expression of SMARCA2 (also called BRM) in neurons [54] and in the liver [55]. In conclusion, data on EMG expression confirmed that the two *in vitro* cultures used here to represent the hepatic and neuronal lineage are more closely related to their respective primary cell types (huHep and Ctx) than to each other.

### Subgroup analysis of differentially expressed EMGs in neurons and hepatocytes

**Figure 5:**
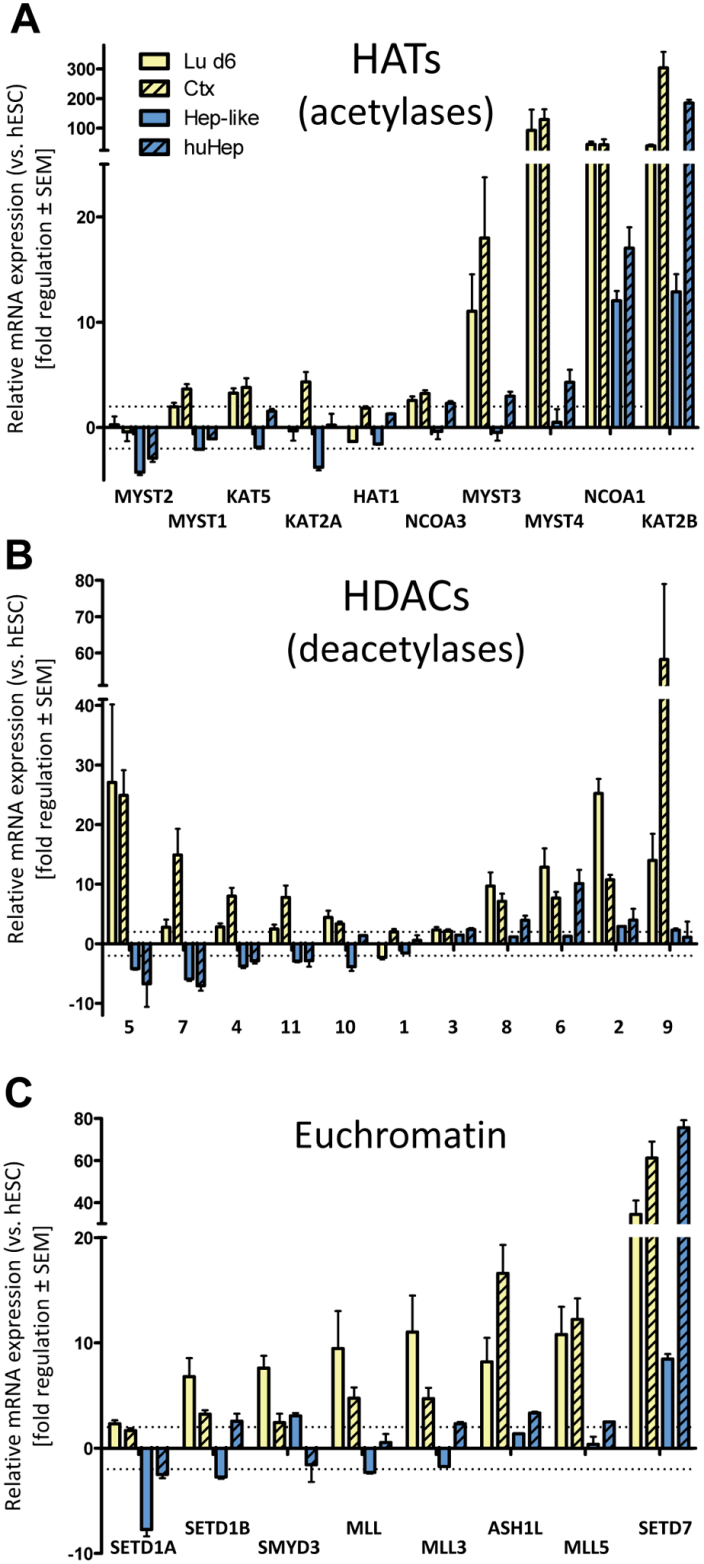
Synopsis of the regulation of euchromatin associated epigenetic modifier groups in different cell lineages. Three groups of epigenetic modifiers were selected for a comparison of relative expression levels of Lu d6, Ctx, Hep-like and huHep. Data were obtained, as described in Fig. 1–3. All data are means ± SEM of three independent differentiations. (**A, B**) Comparison of relative expression levels of histone acetyltransferases (HATs) and histone deacetylases (HDACs) in Lu d6, Ctx, Hep-like and huHep cells. (**C)** Genes responsible for euchromatin establishment and maintenance.

For a more detailed analysis of our data on EMG expression we sorted them according to biological functions. First we took a closer look at histone acetyltransferases (HATs) (Fig. 5A). The majority of HATs showed no or only weak regulation compared to hESC in all cell types. However, two of the ten HATs (NCOA1, KAT2B) were strongly upregulated in all four samples. In addition, two members of the MYST-family (MYST3, 4), which are described to be important for neurogenic progenitor development [56] were strongly upregulated only in the neuronal lineages (Fig. 5A). In contrast, the two other HATs of the same family (MYST1/2) showed no or only a slight upregulation in neurons but downregulation in hepatocytes (Fig. 5A).

Next we compiled an overview of the enzyme class of histone deacetylases (HDACs) (Fig. 5B). HDACs are the counterparts of HATs as they remove acetyl groups from histones and other nuclear and cytoplasmic proteins [57], [58]. We found the HDACs 4/5/7, which all belong to the type IIa class of HDACs [59], to be upregulated in the neuronal but downregulated in the hepatic cell types. HDAC2 and HDAC9 were also upregulated in neuronal cells and not regulated in hepatocytes (Fig. 5B). HDAC1 and HDAC3 were not differentially expressed in comparison to hESC. HDAC1, is known to be ubiquitously expressed in multiple tissues [60] and also plays a central role in ectoderm development [61]. For HDACs a differential expression profile in neurons has been reported [62] and they are a good example for known cell type specific expression of EMGs which has been confirmed by this study. We can assume that cell type specific expression profiles of genes from related protein families might also give clues about the targets and secondary functions of these EMGs.

Even EMGs with an assumed ubiquitous function can show tissue specific expression patterns. This is evident from the examination of euchromatin related genes (Fig. 5C). Most of the genes responsible for euchromatin establishment and maintenance were upregulated in both neuronal cell types relative to their expression levels in hESC. But the hepatic cell types only showed a regulation of two genes (SETD1A/SETD7). SETD1A, a methyltransferase specific for H3K4 and a component of the Set1 complex [63], was downregulated in both hepatic cell types (Fig. 5C). Another methyltransferase of H3K4, SETD7 [64], was upregulated in all four cell types. This suggests a neuronal function of SETD1B and a more general function for SETD7. SETD7 has recently been described to lack specificity for H3K4 and to act also as methyltransferase for non-histone targets [64]. This might explain the ubiquitous expression we observed.

Each cell type needs to organize its genome according to its specific gene expression pattern. One layer of regulation allowing this is the differential expression of EMGs. A good example of EMGs that are regulated in a tissue-specific manner is the group of H3K4-specific SET domain methyltransferases (SETD1A, SETD1B, SMYD3, MLL3, MLL5, SETD7). Almost all of them were highly expressed in the neuronal lineage but not in the hepatic cell types.

**Figure 6:**
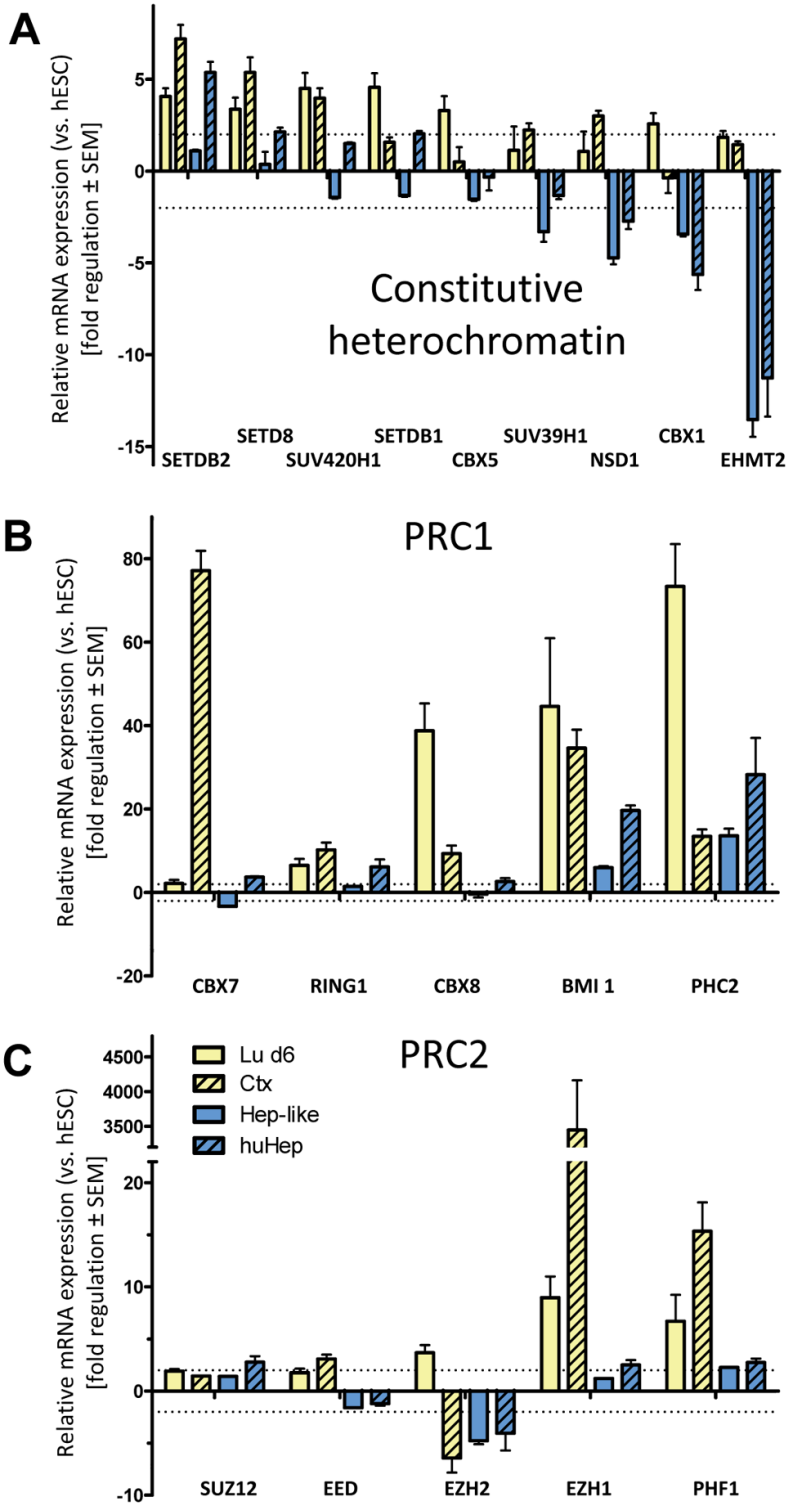
Synopsis of the regulation of heterochromatin associated epigenetic modifier groups in different cell lineages. Three groups of epigenetic modifiers were selected for a comparison of relative expression levels of Lu d6, Ctx, Hep-like and huHep. Data were obtained, as described in Fig. 1–3. All data are means ± SEM of three independent differentiations. (**A**) Genes responsible for heterochromatin establishment and maintenance. (**B, C**) Genes that are involved in polycomb complex (PRC1, PRC2) formation.

We also investigated the pattern of regulation for heterochromatin associated genes which differed between the two lineages (Fig. 6A). Whereas one group (SETD8, SUV420H1, SETDB1, CBX5) was upregulated in the neuronal cell types and not regulated in hepatocytes, the other group of genes (SUV39H1, NSD1, CBX1, EHMT2) was only slightly upregulated in neurons but downregulated in hepatocytes. The only exception to this pattern (of higher neuronal than hepatic expression) was the H3K9 methyltransferase SETDB2 [65] (Fig. 6A). Further, we looked at the polycomb group (PcG) associated genes of the polycomb repressive complex 1 and 2 (PRC1/2) (Fig. 6B/C). PcG-protein complexes are crucial for the regulation of cell fate transitions [66], [67], but have also functions in cell cycle regulation, apoptosis, DNA damage repair and even environmental stress response [68], [69], [70], [71]. We saw only little or no differences in the hepatic samples compared to hESC except for an upregulation of BMI1 and PHC2 (PRC1) and a downregulation of EZH2 (PRC2) in both liver cell types. BMI1 is critical for H2A ubiquitylation, has a broad tissue distribution [72], [73] and is important for the self-renewal capacity of somatic stem cells [74]. PHC2 (polyhomeotic homolog 2) co-localizes with BMI1 and also shows overlapping expression patterns [75], [76]. PHC2 and BMI1 were also strongly upregulated in Lu d6 and Ctx (Fig. 6B).

In contrast to the similar expression pattern of polycomb genes in the two hepatocyte populations, we observed some differences in gene regulation between the two neuronal cell types. Whereas CBX8 was upregulated in differentiated Lu d6 cells, it was not regulated in Ctx tissue (Fig. 6B). An inverse regulation was observed with CBX7. Those two proteins have the same function and substitute each other in a large protein complex [77]. EZH1 and 2 (PRC2) also showed a similar opposite regulation in Ctx and Lu d6 samples. While EZH2 was downregulated, EZH1 was upregulated in Ctx compared Lu d6 cells (Fig. 6C). This is consistent with the important role of EZH2 in progenitor self-renewal [78] and the more abundant expression of EZH1 in adult tissues [79]. This type of regulation might reflect a developmental stage-specific switch in expression of genes with overlapping function. For both of the two PcG complexes PRC1 and PRC2 a switch in the subunit composition of the complex during differentiation from neural progenitor to adult neuron has been described. In the PRC2 of mice the H3K27 methyltransferase EZH2 is partially replaced by EZH1 [78], [79], whereas in PRC1 CBX8 is exchanged for CBX7 [77]. A similar developmental stage-dependent switch has been identified earlier when stem cell derived neural progenitor cells were compared to Ctx [18]. This underlines the usefulness of a comparative approach for identifying cell type and developmental stage-specific regulatory patterns in the expression of EMGs.

Through our comparative approach we also found EMGs that were solely expressed in either the mature or the non-dividing cell types. The relative upregulation of SMARCA2 and BAF45A/53A in both mature cell types agrees with the known subunit switch in certain BAF complexes [16]. Expression patterns of developmentally regulated EMGs may be used for determining the maturity of e.g. differentiating stem cell cultures. Other genes like CBX7, SETD1B, SETD6 and PCGF5 show the same regulation and could represent further maturity markers. The differential gene regulation we observed between cell lineages and also between certain developmental states is well in accordance with previous reports that classified cellular differentiation stages through different chromatin states [80].

### Overview of differentially expressed EMGs

In this study we used four cell types of two different cellular lineages to provide a hitherto unavailable comprehensive overview of the expression patterns of a set of epigenetic modifier genes. Previously, we identified differential expression patterns of epigenetic modifier genes in two neural differentiation systems. Now, we set out to enlarge the scope of the previous study from neural to other cell types. Our aim was to provide complementary information to data on histone modifications, on DNA methylation and on chromatin structure in general. We found that the astonishing diversity of EMG expression patterns between cells adds a layer of information that is not obtainable by other approaches. Of the 156 genes we profiled in this study at least two-thirds were differentially regulated in comparison to hESC even after FDR-correction (Fig. S1, S2). This level of sensitivity cannot be reached by other methods like e.g. microarray analysis.

**Figure 7:**
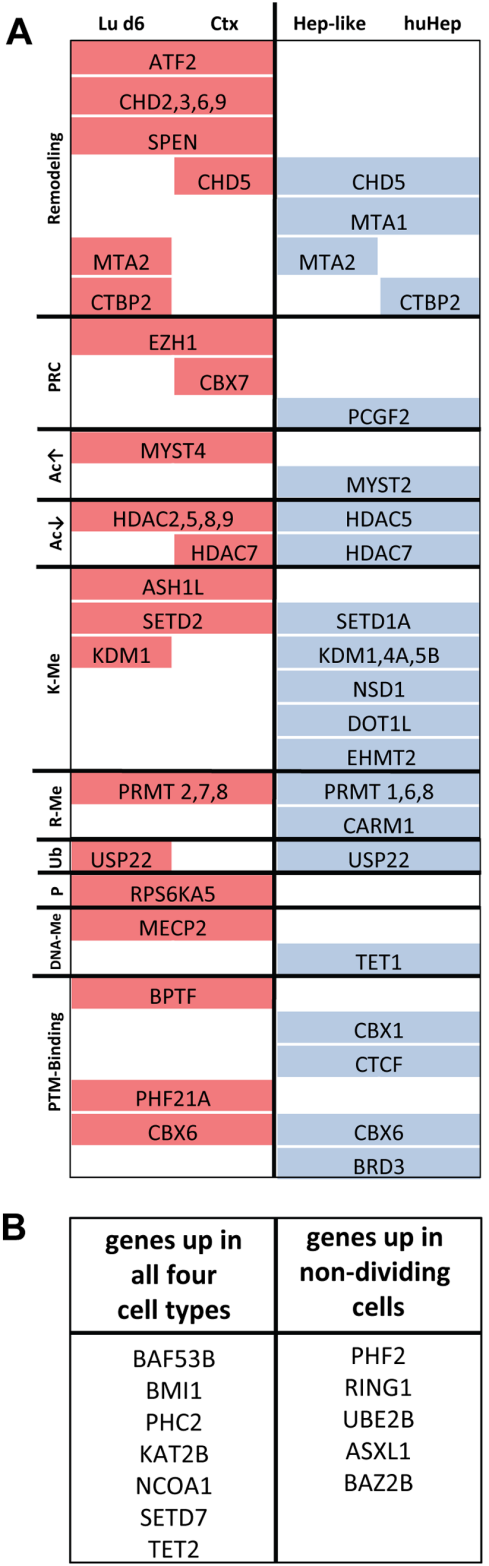
Overview of differentially regulated genetic modifier genes across cell types. (**A**) Original data as displayed in figure 4A were used to compile differentially expressed genes that agree with one tissue type (e.g. hepatic) but not with the other (e.g. neuronal). Gene expression levels higher than in hESC are shown in red, lower levels in blue. Sorting was performed according to biological function (as indicated on the left). PRC: polycomb repressive complex; Ac: acetylation; K-Me: lysine methylation; R-Me: arginine methylation; Ub: ubiquitination; P: phosphorylation; DNA-Me: DNA methylation; PTM: post-translational modification. (**B**) List of genes, whose transcripts are either increased in all 4 cell types compared to hESC (left) or that are upregulated in non-dividing cells (Lu6, Ctx, huHep) as compared to stem cell progeny (right).

In order to summarize our main findings we classified the genes according to their chromatin function and prepared a synoptic overview. Only the most differentially expressed EMGs are shown (Fig. 7A). Some entire gene families showed a cell type specific distribution (e.g. PRMTs). The most conspicuous feature of this overview was that all genes that were differentially regulated between the two cell lineages were upregulated in the neuronal and downregulated in the hepatic cell types. As potential explanation, we considered irregularities with the data normalization procedure. However, this was unlikely, since we normalized to multiple reference genes which showed no differences in expression between cell types or compared to hESC. Another possible explanation could be a fundamental difference in cell response regulation between those two cell types. Liver cells are known to adapt their metabolism and cell function by allosteric regulation and by direct transcriptional regulation through hormones and lipid mediators [81], [82]. Therefore, they might not require strong chromatin dynamics orchestrated by EMGs. In a second summary table (Fig. 7B) we compiled those genes which showed a high expression (compared to hESC) in all four cell types (e.g. BMI1 or PHC2) or in the three non-proliferating cells (Ctx, Lu6, huHep), like the ubiquityltransferase RING1 of the PRC1 complex [67].

Although the understanding of chromatin composition and structure is constantly widening through new methods and model systems (e.g. ChIP-Seq), little is known about the expression patterns of the set of genes that brings about those changes. Chromatin immunoprecipitation, followed by whole genome sequencing techniques can identify binding sites of transcription factors or sites enriched for certain histone modifications [45], [83], but this does not provide information on the respective proteins and complexes that bring about these interactions. Most available studies on epigenetic remodeling complexes and their enzymatic function are limited to mouse or cancer cell models [64], [84].

The set of EMGs compiled here can now help to study human tissue expression of those genes, and to elucidate their possible functional and tissue specific roles. Our approach of combining primary cells (for elucidation of tissue-specific expression profiles) and cells generated in vitro according to various differentiation protocols (easy access to cell material), proved to be a valuable tool for a fast and easy screening of the most important EMGs. However, it needs to be noted that our findings are based on the differentiation of H9 hESC only. There remains the possibility that generation of cells from other pluripotent cel sources may yield different results. Moreover, our conclusions are limited by the low number of tissues and cell types compared here. However, future studies will reveal if our approach can be generalized or the results are specific for the used tissues. Based on our findings that the two stem/precursor cell-derived systems were robust and have a high similarity to primary cultures, they can be used in further mechanistic studies and for a verification of the expression data on protein level.

In conclusion, our study shows that the detection of expression levels of EMGs may be used to classify cell types and their developmental stages. Profiling of EMG expression may also prove to be a useful tool in discerning healthy from diseased tissue states or for distinguishing normal and disturbed differentiation of stem cells [85]. Profiling of EMG expression could provide reliable measures of cell state in addition to classical approaches based on cell function-specific markers or sets of transcription factors.

## Supporting Information

Figure S1 **Fold regulation of EMGs in Lu d6 and Ctx.**
(PDF)

Figure S2 **Fold regulation of EMGs in Hep-like and huHep.**
(PDF)

Figure S3 **Absolute expression data of all cell types and tissues.** Gene expression for 156 EMG and for three houskeeping genes was determinded by RT-qPCR. The threshold cycle values (Ct) were determined with the CFX96 optical system software (Bio-Rad).The geometric mean of three reference genes (HPRT1/RPL13A/GAPDH) was determined (CtRG) and subtracted from the Ct values of the genes of interest. The data (Ct - CtRG) shown here represent the mean of three independent experiments and the standard deviation (SD) is given for each gene.
(PDF)

Figure S4 **Expression of epigenetic regulators genes in LU d6 relative to d0. (A)** RNA was prepared from proliferating LUHMES cells (d0), or the same cells after six days of neuronal differentiation (d6). EMG expression was determined by means of RT-qPCR. The threshold cycle values were determinded with the CFX96 optical system software. Relative gene expression values were calculated by normalization to house keeping genes. Lu d6 data were normalized to d0 expressions (the latter set to 1). Data are means of three independent differentiation. **(B)** p-values were calculated with the SAB online analysis tool and correspond to the statistical difference from the expression levels in Lu d0. SEM were calculated by GraphPad Prism Software.
(PDF)

Figure S5 **Comparison of Ctx and Lu6 gene expression with mesencephalic tissue gene array data and iPSC derived dopaminergic neurons.** Data from a study on dopaminergic neurons (DA d42) that were derived from induced pluripotent stem cells (iPSC) and fetal mesencephalon tissue (huMES) were retrieved from a data base and compared to Lu d6 and Ctx [31]. Microarray data (GSE51214) from this work were normalized to pluripotent stem cells. Data for the 156 EMGs were obstained from this data set. All expression values are given as log2 fold-change values compared to the prespective reference cell source (pluripotent stem cells). A heat map was generated for visualization. The heat map depicts every log fold change <1 with N for “not regulated”, as we defined a cutoff of 2-fold expression changes (1 on the log2 scale).
(PDF)

Table S1 (XLSX)
