# Supplementary material for: Lineage-Specific Regulation of Epigenetic Modifier Genes in Human Liver and Brain
Source: PLoS One. 2014 Jul 23;9(7):e102035. doi: 10.1371/journal.pone.0102035 (PMC4108363; doi:10.1371/journal.pone.0102035)
Supplement: Figure S1 — Fold regulation of EMGs in Lu d6 and Ctx. (PDF) [file pone.0102035.s001.pdf]

Figure S1: Fold regulation of EMGs in Lu d6 and Ctx

gene	Lu d6	SD	p-value	q-value	Ctx	SD	p-value	q-value
ARID1A	5.8	3.1	0.021	0.038	1.0	2.1	0.206	0.225
ASH1L	8.2	3.9	0.012	0.030	16.6	4.7	0.002	0.008
ASXL1	26.8	23.5	0.014	0.029	29.9	1.9	0.000	0.001
ATF2	7.4	3.0	0.007	0.029	11.5	2.3	0.001	0.007
AURKA	-2.5	1.4	0.073	0.093	-1.9	0.6	0.051	0.067
AURKB	-24.0	13.4	0.009	0.031	-17.5	1.7	0.000	0.002
AURKC	0.5	2.1	0.414	0.418	1.5	0.6	0.128	0.148
BAF45A	1.9	1.2	0.147	0.164	23.9	7.6	0.001	0.005
BAF53A	2.4	1.0	0.039	0.058	34.5	22.0	0.006	0.013
BAF53B	6891.0	3743.4	0.001	0.012	1115.2	677.6	0.003	0.008
BAF60A	3.2	1.9	0.087	0.107	4.7	1.4	0.010	0.019
BAF60C	26.2	16.2	0.006	0.028	911.0	119.9	0.000	0.002
BAZ1A	23.0	8.3	0.003	0.020	7.4	2.5	0.005	0.012
BAZ1B	3.3	1.0	0.009	0.031	1.5	0.6	0.109	0.127
BAZ2A	4.1	1.4	0.015	0.030	1.6	0.5	0.071	0.087
BAZ2B	20.5	16.5	0.013	0.030	13.7	4.6	0.001	0.005
BMI1	44.6	28.3	0.006	0.026	34.6	7.6	0.000	0.003
BPTF	4.6	1.9	0.013	0.029	5.4	2.8	0.010	0.019
BRD1	8.7	2.0	0.001	0.013	1.9	0.4	0.016	0.026
BRD2	2.2	0.8	0.050	0.070	1.5	0.2	0.024	0.034
BRD3	3.7	1.3	0.020	0.036	-2.6	0.9	0.033	0.045
BRD4	3.6	2.2	0.039	0.059	1.2	0.1	0.294	0.313
BRD7	1.6	0.5	0.088	0.107	2.4	0.9	0.019	0.030
BRD8	3.9	2.1	0.034	0.051	4.3	0.8	0.002	0.007
BRDT	-2.5	3.4	0.138	0.158	2.2	1.7	0.194	0.215
BRPF1	4.9	0.8	0.010	0.030	2.9	0.6	0.011	0.019
BRPF3	3.2	2.0	0.075	0.095	2.0	0.2	0.012	0.021
BRWD1	5.0	4.0	0.055	0.075	6.7	0.5	0.000	0.002
CARM1	2.0	0.4	0.020	0.036	-1.8	0.3	0.018	0.028
CBX1	2.6	1.0	0.031	0.048	-0.4	1.5	0.434	0.441
CBX3	2.8	0.7	0.011	0.030	-1.4	0.2	0.022	0.032
CBX4	11.4	4.3	0.007	0.026	6.0	0.5	0.002	0.007
CBX5	3.3	1.3	0.026	0.044	0.5	1.5	0.257	0.276
CBX6	6.4	2.6	0.010	0.030	11.9	2.7	0.000	0.004
CBX7	2.1	1.5	0.123	0.145	77.1	8.3	0.000	0.002
CBX8	38.8	11.4	0.000	0.012	9.3	3.3	0.002	0.007
CDYL	-1.6	0.1	0.013	0.029	-1.4	0.3	0.080	0.098
CDYL2	4.3	3.5	0.080	0.099	23.5	7.8	0.001	0.004
CHD1	3.7	1.9	0.039	0.058	2.6	0.6	0.009	0.018
CHD2	5.1	4.1	0.052	0.071	4.6	1.0	0.004	0.011
CHD3	14.5	10.7	0.017	0.033	4.3	0.4	0.001	0.005
CHD4	8.2	5.3	0.013	0.030	2.3	1.0	0.051	0.067
CHD5	1.7	3.5	0.270	0.279	83.8	47.9	0.002	0.007
CHD6	5.3	1.0	0.002	0.017	4.4	2.0	0.011	0.019
CHD7	8.2	4.2	0.009	0.030	3.2	0.4	0.000	0.004
CHD8	4.0	2.6	0.052	0.071	2.6	0.4	0.004	0.010
CHD9	13.7	4.9	0.004	0.021	9.1	1.2	0.000	0.003
CSRP2BP	2.3	0.4	0.004	0.022	1.0	2.0	0.168	0.188
CTBP1	3.8	1.4	0.018	0.035	4.3	0.3	0.000	0.003
CTBP2	4.6	2.4	0.022	0.038	1.7	0.5	0.025	0.036
CTCF	1.9	0.7	0.061	0.080	0.6	1.5	0.241	0.261
DNMT1	0.8	1.7	0.219	0.229	10.7	10.6	0.109	0.128
DNMT3A	3.1	1.4	0.028	0.045	-3.2	1.5	0.027	0.039
DNMT3B	-19.0	7.1	0.004	0.022	-336.7	218.0	0.002	0.007
DOT1L	3.3	2.1	0.044	0.063	-1.3	2.3	0.160	0.180
DZIP3	4.0	2.3	0.031	0.048	3.9	1.6	0.016	0.026
EED	1.8	0.7	0.080	0.099	3.1	0.7	0.008	0.016
EHMT2	1.8	0.6	0.049	0.069	1.4	0.3	0.058	0.073

gene	Lu d6	SD	p-value	q-value	Ctx	SD	p-value	q-value
ESCO1	1.9	1.1	0.131	0.152	2.2	0.6	0.014	0.024
ESCO2	-15.9	2.2	0.000	0.018	-14.4	2.4	0.001	0.005
EZH1	9.0	3.5	0.006	0.028	3446.5	1244.2	0.000	0.004
EZH2	3.7	1.3	0.014	0.029	-6.5	2.4	0.006	0.013
HAT1	-1.3	0.0	0.024	0.041	1.8	0.3	0.008	0.016
HDAC1	-2.3	0.6	0.011	0.030	2.0	0.9	0.068	0.084
HDAC10	4.4	2.0	0.008	0.031	3.4	0.6	0.005	0.011
HDAC11	2.5	1.3	0.077	0.096	7.8	3.4	0.015	0.025
HDAC2	25.2	4.3	0.004	0.023	10.7	1.4	0.007	0.014
HDAC3	2.3	0.8	0.026	0.044	2.1	0.5	0.019	0.029
HDAC4	2.8	1.0	0.022	0.038	8.0	2.4	0.022	0.032
HDAC5	27.1	22.7	0.011	0.031	24.9	7.4	0.001	0.007
HDAC6	12.9	5.5	0.006	0.028	7.6	1.8	0.003	0.008
HDAC7	2.8	2.2	0.139	0.157	14.9	7.6	0.010	0.020
HDAC8	9.7	4.0	0.002	0.019	7.1	2.3	0.002	0.007
HDAC9	14.0	7.7	0.013	0.029	58.2	36.1	0.004	0.009
ING1	2.0	0.8	0.062	0.080	2.9	0.4	0.019	0.029
ING2	1.3	2.4	0.181	0.195	2.6	0.3	0.002	0.007
ING3	5.0	1.1	0.002	0.020	3.7	0.7	0.002	0.008
ING4	12.4	3.3	0.001	0.015	3.7	0.2	0.005	0.011
ING5	1.0	1.8	0.158	0.173	2.7	0.3	0.001	0.007
INO80	3.0	1.7	0.059	0.079	2.6	0.3	0.002	0.007
KAT2A	-0.3	1.6	0.500	0.500	4.3	1.6	0.015	0.025
KAT2B	40.4	6.9	0.000	0.014	303.6	93.6	0.001	0.005
KAT5	3.3	0.8	0.008	0.031	3.8	1.5	0.017	0.027
KDM1	4.1	0.9	0.005	0.025	-0.6	1.4	0.158	0.180
KDM4A	2.3	0.6	0.013	0.031	0.3	1.3	0.420	0.430
KDM4C	2.2	1.1	0.069	0.089	2.7	0.3	0.001	0.007
KDM5B	2.7	0.7	0.012	0.031	-2.5	1.2	0.066	0.082
KDM5C	2.8	0.8	0.023	0.039	-1.9	3.3	0.202	0.222
KDM6B	11.3	3.3	0.003	0.021	1.3	0.1	0.012	0.021
MBD1	4.0	2.0	0.029	0.046	2.7	0.3	0.001	0.005
MBD2	4.1	1.5	0.014	0.030	4.9	1.0	0.003	0.007
MBD3	2.4	0.8	0.040	0.058	3.1	0.7	0.003	0.009
MBD4	1.1	1.9	0.127	0.149	3.2	0.5	0.004	0.009
MECP2	22.9	8.1	0.003	0.020	28.0	3.0	0.000	0.004
MLL	9.5	6.2	0.022	0.038	4.7	1.8	0.018	0.028
MLL3	11.0	6.0	0.013	0.029	4.7	1.8	0.015	0.024
MLL5	10.8	4.6	0.006	0.027	12.2	3.5	0.002	0.007
MTA1	1.1	1.9	0.129	0.150	-1.2	0.2	0.150	0.171
MTA2	4.7	1.8	0.006	0.029	0.8	1.7	0.226	0.246
MYSM1	1.0	2.0	0.212	0.225	2.1	0.5	0.012	0.021
MYST1	2.0	0.7	0.044	0.063	3.6	0.8	0.002	0.008
MYST2	0.3	1.4	0.421	0.424	-0.4	1.5	0.435	0.440
MYST3	11.0	6.1	0.013	0.029	18.0	10.0	0.002	0.007
MYST4	92.4	121.5	0.020	0.036	128.8	60.9	0.002	0.007
NCOA1	45.0	16.4	0.001	0.012	43.7	33.0	0.003	0.008
NCOA3	2.6	0.7	0.006	0.028	3.2	0.5	0.002	0.007
NEK6	1.0	1.8	0.137	0.158	12.9	5.3	0.006	0.013
NSD1	1.0	1.9	0.142	0.160	3.0	0.5	0.001	0.007
PAK1	0.7	1.5	0.155	0.171	-0.5	2.0	0.397	0.411
PBRM1	17.2	10.5	0.009	0.031	4.3	0.6	0.000	0.004
PCGF1	4.3	1.5	0.010	0.030	3.6	0.5	0.002	0.007
PCGF2	3.7	1.9	0.028	0.045	1.4	0.2	0.041	0.055
PCGF5	-6.3	1.2	0.001	0.013	43.4	9.3	0.000	0.006
PHC1	-3.7	1.6	0.010	0.030	-8.0	3.5	0.003	0.009
PHC2	73.3	17.6	0.000	0.018	13.5	2.9	0.001	0.005
PHF1	6.7	4.4	0.029	0.045	15.3	4.8	0.016	0.026
PHF13	1.2	2.2	0.195	0.210	-1.6	0.2	0.065	0.082
PHF2	10.6	1.8	0.001	0.015	14.7	4.0	0.001	0.005
PHF21A	7.9	5.3	0.010	0.031	5.7	1.7	0.004	0.010

gene	Lu d6	SD	p-value	q-value	Ctx	SD	p-value	q-value
PRMT1	1.8	0.4	0.018	0.035	-1.7	0.2	0.012	0.021
PRMT2	7.1	3.2	0.011	0.031	5.8	1.4	0.004	0.011
PRMT3	-0.4	1.3	0.362	0.370	1.3	2.1	0.109	0.128
PRMT5	1.2	0.0	0.148	0.163	-0.3	1.7	0.489	0.492
PRMT6	2.0	0.3	0.005	0.026	0.7	2.3	0.334	0.352
PRMT7	4.5	2.9	0.027	0.044	5.4	1.8	0.006	0.013
PRMT8	8.0	6.6	0.060	0.079	28.5	24.0	0.012	0.021
RING1	6.5	2.7	0.003	0.022	10.2	3.0	0.001	0.006
RNF2	2.8	0.3	0.002	0.018	0.4	1.4	0.324	0.343
RNF20	3.5	0.1	0.002	0.019	4.3	1.5	0.011	0.020
RPS6KA3	2.0	0.8	0.073	0.094	20.1	4.6	0.001	0.005
RPS6KA5	4.8	3.5	0.061	0.080	28.5	7.3	0.000	0.003
SETD1A	2.3	0.6	0.018	0.034	1.6	0.4	0.046	0.060
SETD1B	6.8	3.0	0.011	0.030	3.3	0.6	0.002	0.008
SETD2	4.9	1.9	0.013	0.030	6.2	2.7	0.010	0.019
SETD3	2.1	0.5	0.019	0.036	5.4	2.0	0.009	0.018
SETD6	3.9	1.7	0.021	0.037	2.4	0.7	0.021	0.031
SETD7	34.4	11.5	0.000	0.018	61.2	13.5	0.000	0.009
SETD8	3.4	1.1	0.014	0.030	5.4	1.5	0.005	0.012
SETDB1	4.6	1.3	0.006	0.028	1.5	0.5	0.074	0.091
SETDB2	4.1	0.8	0.001	0.018	7.2	1.3	0.000	0.004
SMARCA2	3.2	2.1	0.096	0.115	78.4	14.3	0.000	0.018
SMARCA4	6.8	4.2	0.017	0.033	1.4	0.1	0.188	0.209
SMYD3	7.6	2.0	0.002	0.019	2.4	1.4	0.063	0.079
SPEN	6.0	4.0	0.027	0.044	5.6	1.8	0.003	0.008
SUV39H1	1.1	2.3	0.213	0.225	2.2	0.6	0.028	0.039
SUV420H1	4.5	1.4	0.009	0.031	4.0	1.0	0.006	0.013
SUZ12	1.9	0.3	0.010	0.031	1.5	0.0	0.028	0.039
TET1	3.2	2.6	0.104	0.123	0.5	1.6	0.413	0.425
TET2	80.7	6.2	0.002	0.017	101.8	5.8	0.002	0.007
UBE2A	4.1	1.7	0.012	0.031	9.3	3.2	0.002	0.007
UBE2B	4.7	0.9	0.002	0.020	7.7	1.3	0.001	0.006
USP16	3.4	1.3	0.019	0.035	11.1	3.9	0.005	0.011
USP21	4.0	2.7	0.040	0.059	8.5	2.5	0.004	0.010
USP22	4.6	1.9	0.016	0.033	1.6	0.3	0.034	0.046
WHSC1	5.8	2.2	0.010	0.031	2.7	1.0	0.025	0.036
