# Supplementary material for: Lineage-Specific Regulation of Epigenetic Modifier Genes in Human Liver and Brain
Source: PLoS One. 2014 Jul 23;9(7):e102035. doi: 10.1371/journal.pone.0102035 (PMC4108363; doi:10.1371/journal.pone.0102035)
Supplement: Figure S2 — Fold regulation of EMGs in Hep-like and huHep. (PDF) [file pone.0102035.s002.pdf]

Figure S2: Fold regulation of EMGs in Hep-like and huHep

gene	Hep-like	SD	p-value	q-value	huHep	SD	p-value	q-value
ARID1A	-2.6	0.7	0.007	0.016	-3.4	2.6	0.076	0.102
ASH1L	1.4	0.1	0.006	0.016	3.3	0.2	0.000	0.003
ASXL1	2.2	0.1	0.004	0.012	5.6	1.1	0.001	0.006
ATF2	-1.1	0.1	0.098	0.121	2.8	0.2	0.000	0.005
AURKA	-4.0	0.1	0.000	0.004	-4.6	1.6	0.009	0.022
AURKB	-8.4	0.5	0.001	0.007	-41.6	11.8	0.000	0.004
AURKC	-4.0	0.6	0.000	0.005	1.4	0.3	0.052	0.075
BAF45A	2.2	0.6	0.037	0.051	5.3	2.1	0.007	0.018
BAF53A	5.9	1.0	0.000	0.005	11.5	0.7	0.001	0.005
BAF53B	5.4	3.2	0.046	0.062	37.0	26.2	0.018	0.034
BAF60A	-2.7	0.1	0.003	0.011	-2.8	0.4	0.002	0.009
BAF60C	39.8	22.8	0.004	0.013	-1.8	3.1	0.185	0.212
BAZ1A	2.3	0.1	0.002	0.008	7.6	1.2	0.001	0.007
BAZ1B	-2.6	0.6	0.006	0.016	-0.7	1.6	0.185	0.212
BAZ2A	-2.9	0.4	0.002	0.008	-2.5	0.8	0.028	0.046
BAZ2B	1.8	0.5	0.067	0.086	6.3	1.1	0.000	0.005
BMI1	6.0	0.6	0.004	0.012	19.6	2.1	0.002	0.007
BPTF	-1.4	0.1	0.129	0.153	1.5	0.2	0.115	0.140
BRD1	-2.3	0.3	0.003	0.011	-0.5	1.5	0.319	0.342
BRD2	-1.8	0.2	0.010	0.019	1.9	0.2	0.007	0.018
BRD3	-7.6	2.4	0.001	0.007	-6.0	3.7	0.022	0.039
BRD4	-1.6	0.3	0.107	0.130	1.6	0.5	0.106	0.130
BRD7	-1.5	0.1	0.051	0.067	0.5	1.4	0.327	0.349
BRD8	-3.3	0.2	0.007	0.016	1.4	0.2	0.084	0.110
BRDT	-21.6	10.8	0.006	0.016	-1.8	0.3	0.143	0.171
BRPF1	-2.8	0.6	0.012	0.021	-2.1	1.3	0.123	0.149
BRPF3	-1.8	0.3	0.014	0.023	-0.3	1.4	0.434	0.446
BRWD1	-1.5	0.2	0.016	0.026	3.0	0.2	0.000	0.006
CARM1	-5.5	0.4	0.000	0.004	-5.8	1.8	0.005	0.015
CBX1	-3.4	0.2	0.009	0.018	-5.6	1.5	0.002	0.010
CBX3	-0.4	1.3	0.198	0.222	1.3	0.2	0.073	0.098
CBX4	1.4	0.2	0.047	0.063	5.4	5.0	0.054	0.077
CBX5	-1.5	0.2	0.019	0.030	-0.4	1.2	0.435	0.446
CBX6	-2.8	1.1	0.031	0.045	-13.0	15.4	0.052	0.075
CBX7	-3.3	0.1	0.001	0.005	3.7	0.3	0.000	0.004
CBX8	-0.4	1.4	0.393	0.398	2.6	1.4	0.055	0.077
CDYL	-2.2	0.2	0.002	0.008	-5.1	0.2	0.001	0.007
CDYL2	2.1	0.3	0.006	0.016	7.9	1.9	0.001	0.006
CHD1	1.6	0.1	0.029	0.042	5.2	2.7	0.018	0.034
CHD2	0.4	1.3	0.239	0.261	2.4	0.3	0.001	0.007
CHD3	0.5	1.3	0.168	0.190	-3.8	0.7	0.002	0.008
CHD4	-2.5	0.2	0.029	0.042	-1.9	0.2	0.058	0.079
CHD5	-141.0	23.9	0.002	0.008	-97.4	66.8	0.011	0.025
CHD6	-1.3	0.1	0.150	0.172	2.6	0.8	0.016	0.032
CHD7	-1.5	0.1	0.004	0.012	-0.6	1.4	0.233	0.256
CHD8	-3.0	0.3	0.007	0.016	-2.1	0.5	0.019	0.034
CHD9	1.6	0.1	0.010	0.019	3.2	0.7	0.007	0.019
CSRP2BP	-1.3	0.1	0.034	0.049	1.2	0.2	0.141	0.169
CTBP1	-1.8	0.2	0.004	0.013	0.5	1.4	0.247	0.270
CTBP2	-2.8	0.3	0.001	0.007	-28.4	20.3	0.009	0.022
CTCF	-4.6	0.2	0.002	0.008	-249.1	428.1	0.157	0.186
DNMT1	-4.6	0.3	0.010	0.019	-3.2	0.9	0.012	0.026
DNMT3A	-6.2	0.5	0.000	0.004	-17.6	6.6	0.003	0.011
DNMT3B	-106.8	20.6	0.000	0.004	-405.0	123.3	0.001	0.006
DOT1L	-7.4	0.8	0.001	0.005	-4.4	0.9	0.003	0.010
DZIP3	-2.2	0.2	0.002	0.008	-2.4	0.2	0.001	0.007
EED	-1.6	0.1	0.001	0.008	-10.4	15.8	0.180	0.209
EHMT2	-13.5	1.6	0.000	0.006	-11.3	3.6	0.004	0.013

gene	Hep-like	SD	p-value	q-value	huHep	SD	p-value	q-value
ESCO1	0.4	1.2	0.436	0.436	1.8	0.1	0.019	0.035
ESCO2	-8.4	1.6	0.002	0.008	-9.2	4.1	0.008	0.021
EZH1	1.2	0.1	0.301	0.313	2.5	0.8	0.048	0.071
EZH2	-4.8	0.6	0.010	0.019	-25.9	37.8	0.078	0.104
HAT1	-1.6	0.1	0.005	0.015	1.3	0.1	0.029	0.048
HDAC1	-1.5	0.1	0.030	0.044	0.6	1.5	0.205	0.229
HDAC10	-3.8	1.3	0.007	0.016	1.4	0.1	0.097	0.122
HDAC11	-2.9	0.4	0.013	0.022	-2.8	1.8	0.084	0.110
HDAC2	2.9	0.2	0.028	0.042	4.0	3.3	0.097	0.123
HDAC3	1.5	0.1	0.011	0.020	2.4	0.5	0.011	0.025
HDAC4	-3.7	0.6	0.001	0.006	-2.9	0.8	0.016	0.032
HDAC5	-4.2	0.3	0.000	0.009	-6.7	6.7	0.056	0.077
HDAC6	1.3	0.1	0.029	0.042	10.1	3.9	0.005	0.015
HDAC7	-5.9	0.6	0.028	0.042	-7.0	1.4	0.025	0.043
HDAC8	1.1	0.0	0.209	0.232	3.9	1.4	0.005	0.015
HDAC9	2.3	0.4	0.012	0.021	1.1	4.6	0.378	0.394
ING1	-1.6	0.1	0.066	0.086	0.7	2.7	0.369	0.387
ING2	-1.3	0.2	0.035	0.050	0.1	2.4	0.454	0.462
ING3	1.3	0.1	0.100	0.123	3.1	1.3	0.040	0.062
ING4	-0.6	1.4	0.145	0.168	2.0	0.8	0.092	0.118
ING5	-3.5	0.6	0.001	0.008	-1.5	0.3	0.033	0.053
INO80	-2.6	0.7	0.005	0.014	0.5	1.4	0.179	0.209
KAT2A	-3.8	0.5	0.002	0.008	0.2	1.9	0.455	0.460
KAT2B	12.9	2.9	0.002	0.009	184.7	19.1	0.000	0.004
KAT5	-1.9	0.3	0.009	0.018	1.5	0.4	0.057	0.079
KDM1	-3.5	0.2	0.000	0.003	-5.2	1.4	0.005	0.015
KDM4A	-6.5	1.0	0.001	0.006	-2.2	0.6	0.020	0.035
KDM4C	-1.1	0.0	0.171	0.193	3.2	0.4	0.001	0.007
KDM5B	-1.6	0.2	0.010	0.019	-7.5	3.5	0.011	0.025
KDM5C	-1.5	0.2	0.133	0.158	-2.8	0.8	0.020	0.036
KDM6B	-1.5	0.1	0.007	0.016	-0.6	1.5	0.222	0.245
MBD1	-2.4	0.4	0.008	0.018	-1.6	2.4	0.103	0.128
MBD2	1.4	0.1	0.007	0.016	3.5	0.9	0.006	0.007
MBD3	-2.5	0.7	0.007	0.016	-0.5	1.7	0.332	0.350
MBD4	-1.1	0.1	0.088	0.111	2.5	0.7	0.017	0.033
MECP2	1.2	0.1	0.014	0.023	-0.3	1.4	0.467	0.470
MLL	-2.3	0.2	0.012	0.021	0.5	1.4	0.219	0.244
MLL3	-1.7	0.0	0.005	0.014	2.4	0.3	0.002	0.007
MLL5	0.4	1.3	0.290	0.306	2.5	0.0	0.002	0.009
MTA1	-5.6	1.5	0.002	0.009	-5.9	2.0	0.002	0.010
MTA2	-6.7	1.0	0.005	0.015	-3.2	0.1	0.011	0.025
MYSM1	-2.5	0.4	0.003	0.012	-1.1	0.1	0.161	0.190
MYST1	-2.1	0.1	0.008	0.017	-1.1	0.1	0.270	0.294
MYST2	-4.2	0.5	0.013	0.022	-2.9	0.6	0.027	0.045
MYST3	-0.5	1.3	0.285	0.304	3.0	0.7	0.009	0.022
MYST4	0.5	2.2	0.389	0.396	4.3	2.1	0.033	0.052
NCOA1	12.0	1.6	0.007	0.016	17.0	3.4	0.006	0.017
NCOA3	-0.4	1.3	0.373	0.381	2.3	0.4	0.003	0.012
NEK6	2.2	0.2	0.002	0.008	8.0	3.5	0.008	0.019
NSD1	-4.7	0.6	0.000	0.005	-2.7	0.8	0.018	0.034
PAK1	-3.3	0.3	0.006	0.017	-4.1	0.6	0.001	0.007
PBRM1	-1.1	0.1	0.121	0.145	3.4	0.4	0.001	0.006
PCGF1	0.4	1.3	0.284	0.306	2.1	0.5	0.016	0.032
PCGF2	-2.0	0.3	0.006	0.016	-6.1	1.3	0.001	0.006
PCGF5	4.6	0.4	0.004	0.013	38.4	8.5	0.000	0.006
PHC1	-43.5	6.8	0.000	0.018	-94.4	23.2	0.000	0.009
PHC2	13.6	3.0	0.001	0.007	28.2	15.3	0.002	0.009
PHF1	2.3	0.1	0.108	0.131	2.7	0.6	0.086	0.112
PHF13	-3.4	0.5	0.012	0.021	-2.3	0.5	0.015	0.031
PHF2	2.8	0.6	0.007	0.016	6.9	1.7	0.001	0.007
PHF21A	-1.6	0.2	0.086	0.109	-1.9	0.2	0.052	0.075

gene	Hep-like	SD	p-value	q-value	huHep	SD	p-value	q-value
PRMT1	-4.4	0.1	0.002	0.009	-6.8	0.9	0.000	0.006
PRMT2	0.3	1.2	0.286	0.303	-1.4	0.2	0.048	0.071
PRMT3	-2.2	0.2	0.024	0.036	-0.4	1.3	0.412	0.426
PRMT5	-1.2	0.1	0.139	0.163	-1.2	0.1	0.177	0.208
PRMT6	-6.3	3.1	0.012	0.021	-2.1	0.8	0.039	0.060
PRMT7	1.3	0.0	0.001	0.005	1.8	0.2	0.004	0.014
PRMT8	-169.9	204.3	0.014	0.023	-15.8	12.1	0.036	0.057
RING1	1.5	0.1	0.054	0.070	6.2	3.1	0.015	0.031
RNF2	1.1	0.0	0.282	0.307	-1.8	0.3	0.012	0.027
RNF20	-0.2	1.3	0.285	0.306	2.7	0.3	0.001	0.007
RPS6KA3	7.3	0.5	0.006	0.016	20.5	3.5	0.003	0.011
RPS6KA5	1.3	0.1	0.091	0.114	2.5	1.0	0.017	0.033
SETD1A	-7.7	1.1	0.001	0.007	-2.5	0.6	0.012	0.026
SETD1B	-2.7	0.3	0.002	0.008	2.6	1.2	0.045	0.068
SETD2	-1.8	0.1	0.011	0.020	2.2	0.2	0.002	0.010
SETD3	1.3	0.1	0.002	0.009	2.3	0.1	0.000	0.018
SETD6	-2.0	0.3	0.005	0.014	3.2	0.5	0.001	0.007
SETD7	8.5	0.8	0.003	0.011	75.7	6.1	0.001	0.006
SETD8	0.4	1.2	0.329	0.340	2.2	0.4	0.012	0.027
SETDB1	-1.3	0.1	0.007	0.016	2.0	0.3	0.007	0.018
SETDB2	1.1	0.1	0.227	0.251	5.4	1.0	0.001	0.006
SMARCA2	6.3	0.1	0.002	0.008	53.8	28.2	0.003	0.011
SMARCA4	-3.9	0.8	0.021	0.033	-3.8	0.9	0.011	0.025
SMYD3	3.1	0.5	0.015	0.023	-1.6	2.9	0.202	0.228
SPEN	-2.2	0.4	0.014	0.023	-0.2	4.8	0.482	0.482
SUV39H1	-3.3	0.9	0.009	0.018	-1.4	0.4	0.105	0.131
SUV420H1	-1.4	0.1	0.014	0.022	1.5	0.1	0.001	0.006
SUZ12	1.4	0.0	0.038	0.053	2.7	1.0	0.037	0.058
TET1	-1.8	0.5	0.139	0.162	-79.0	70.5	0.007	0.018
TET2	8.1	1.3	0.010	0.019	27.1	1.7	0.004	0.012
UBE2A	1.7	0.2	0.078	0.100	2.5	0.7	0.024	0.042
UBE2B	1.6	0.0	0.041	0.055	6.3	0.3	0.003	0.012
USP16	1.8	0.0	0.009	0.018	7.1	1.0	0.000	0.005
USP21	-1.6	0.1	0.000	0.004	-1.9	0.2	0.006	0.018
USP22	-4.4	0.6	0.000	0.005	-5.9	2.9	0.016	0.032
WHSC1	-3.5	0.3	0.000	0.005	-2.4	0.2	0.001	0.006
