# Supplementary material for: Lineage-Specific Regulation of Epigenetic Modifier Genes in Human Liver and Brain
Source: PLoS One. 2014 Jul 23;9(7):e102035. doi: 10.1371/journal.pone.0102035 (PMC4108363; doi:10.1371/journal.pone.0102035)
Supplement: Figure S3 — Absolute expression data of all cell types and tissues. Gene expression for 156 EMG and for three houskeeping genes was determinded by RT-qPCR. The threshold cycle values (Ct) were determined with the CFX96 optical system software (Bio-Rad).The geometric mean of three reference genes (HPRT1/RPL13A/GAPDH) was determined (CtRG) and subtracted from the Ct values of the genes of interest. The data (Ct - CtRG) shown here represent the mean of three independent experiments and the standard deviation (SD) is given for each gene. (PDF) [file pone.0102035.s003.pdf]

Figure S3: Absolute expression data of all cell types and tissues

gene	ΔCt H9	SD	ΔCt Lu d0	SD	ΔCt Lu d6	SD	ΔCt Ctx	SD	ΔCt Hep-like	SD	ΔCt huHep	SD
ARID1A	5.65	0.25	4.90	0.55	3.26	0.85	5.09	0.91	7.01	0.38	7.11	1.09
ASH1L	8.39	0.09	7.04	0.48	5.48	0.77	4.37	0.45	7.95	0.10	6.65	0.10
ASXL1	9.02	0.17	6.01	0.79	4.65	1.27	4.12	0.09	7.91	0.04	6.55	0.32
ATF2	5.84	0.13	4.83	0.65	3.02	0.58	2.33	0.27	6.00	0.13	4.34	0.11
AURKA	5.11	0.02	4.43	0.23	6.28	0.86	5.95	0.50	7.13	0.04	7.27	0.50
AURKB	5.05	0.23	3.16	0.51	9.43	0.98	9.17	0.14	8.12	0.08	10.39	0.42
AURKC	10.52	0.12	11.62	1.13	10.37	1.07	10.02	0.54	12.50	0.22	10.02	0.29
BAF45A	6.93	0.55	5.75	0.70	6.19	0.85	2.40	0.50	5.86	0.40	4.60	0.54
BAF53A	7.95	0.20	6.58	0.93	6.75	0.59	3.04	0.92	5.40	0.23	4.43	0.09
BAF53B	16.87	0.17	19.44	1.95	4.25	0.73	7.02	1.20	14.71	1.20	12.11	1.61
BAF60A	5.17	0.20	4.51	0.65	3.73	1.19	2.99	0.49	6.59	0.03	6.62	0.24
BAF60C	11.69	0.14	7.73	0.82	7.15	0.82	1.87	0.20	6.52	0.76	12.56	1.31
BAZ1A	8.38	0.12	3.79	0.34	3.92	0.54	5.55	0.49	7.19	0.05	5.47	0.23
BAZ1B	5.77	0.31	4.61	0.22	4.12	0.52	5.22	0.53	7.14	0.31	6.13	0.51
BAZ2A	5.76	0.22	5.08	0.29	3.80	0.55	5.09	0.44	7.30	0.21	7.01	0.49
BAZ2B	7.95	0.25	7.10	0.59	3.89	1.11	4.22	0.46	7.17	0.49	5.30	0.23
BMI1	8.27	0.37	4.56	0.22	3.00	0.97	3.17	0.30	5.70	0.15	3.98	0.16
BPTF	5.23	0.54	4.89	0.31	3.14	0.69	2.91	0.68	5.74	0.13	4.65	0.24
BRD1	8.22	0.25	7.21	0.08	5.13	0.36	7.34	0.31	9.41	0.16	8.38	0.46
BRD2	5.98	0.26	5.56	0.20	4.94	0.56	5.41	0.15	6.83	0.20	5.04	0.18
BRD3	7.04	0.27	7.20	0.38	5.23	0.58	8.35	0.55	9.92	0.43	9.44	0.87
BRD4	5.56	0.57	4.74	0.47	3.91	0.93	5.35	0.08	6.22	0.26	4.92	0.41
BRD7	5.61	0.34	4.63	0.22	5.00	0.51	4.38	0.50	6.20	0.05	5.48	0.35
BRD8	6.16	0.33	5.18	0.53	4.35	0.79	4.07	0.25	7.87	0.09	5.71	0.16
BRDT	13.77	1.00	16.67	1.45	15.07	1.37	12.93	1.05	18.04	0.92	14.63	0.26
BRPF1	10.19	0.52	9.52	0.35	7.91	0.23	8.65	0.30	11.67	0.31	11.04	0.88
BRPF3	6.72	0.24	7.35	0.05	5.26	1.08	5.74	0.11	7.52	0.24	6.68	0.37
BRWD1	6.88	0.17	6.34	0.81	4.90	1.24	4.14	0.12	7.42	0.18	5.28	0.10
CARM1	4.33	0.12	3.76	0.33	3.32	0.33	5.17	0.26	6.79	0.11	6.84	0.41
CBX1	4.04	0.40	3.60	0.03	2.76	0.66	4.11	0.41	5.81	0.09	6.50	0.38
CBX3	3.54	0.12	2.29	0.33	2.08	0.35	3.97	0.19	3.65	0.15	3.15	0.26
CBX4	9.10	0.25	7.87	0.07	5.68	0.64	6.51	0.11	8.64	0.20	7.06	1.25
CBX5	3.81	0.25	2.59	0.13	2.19	0.62	3.60	0.41	4.42	0.17	3.84	0.13
CBX6	13.20	0.25	10.91	0.46	10.60	0.60	9.66	0.35	14.62	0.60	16.16	1.78
CBX7	10.01	0.10	10.85	0.61	9.12	0.94	3.74	0.15	11.74	0.05	8.12	0.13
CBX8	10.52	0.34	7.05	0.42	5.28	0.40	7.36	0.53	10.59	0.32	9.26	0.71
CDYL	5.03	0.17	5.38	0.19	5.90	0.49	5.57	0.49	6.27	0.20	7.41	0.04
CDYL2	11.02	0.27	15.05	1.06	9.29	1.34	6.52	0.52	9.96	0.18	8.06	0.33
CHD1	6.87	0.27	5.15	0.25	5.15	0.84	5.55	0.40	6.21	0.08	4.61	0.71
CHD2	7.37	0.15	6.35	0.80	5.34	1.22	5.18	0.31	7.22	0.26	6.09	0.15
CHD3	7.53	0.28	6.13	0.29	3.96	1.13	5.42	0.15	7.28	0.25	9.46	0.27
CHD4	3.97	0.56	2.62	0.28	1.16	1.03	2.87	0.60	5.28	0.14	4.87	0.18
CHD5	12.01	0.65	12.29	0.70	11.29	1.56	5.85	1.09	19.14	0.26	18.22	1.50
CHD6	8.15	0.40	7.50	0.14	5.77	0.29	6.12	0.67	8.48	0.12	6.81	0.46
CHD7	7.51	0.10	5.48	0.53	4.60	0.69	5.85	0.16	8.11	0.12	7.75	0.44

gene	ΔCt H9	SD	ΔCt Lu d0	SD	ΔCt Lu d6	SD	ΔCt Ctx	SD	ΔCt Hep-like	SD	ΔCt huHep	SD
CHD8	4.77	0.29	4.59	0.63	3.00	1.03	3.38	0.24	6.33	0.14	5.79	0.40
CHD9	6.48	0.15	4.82	0.31	2.77	0.56	3.31	0.19	5.83	0.07	4.83	0.32
CSRP2BP	7.68	0.19	7.23	0.07	6.49	0.25	7.06	0.84	8.07	0.14	7.47	0.21
CTBP1	4.76	0.16	3.78	0.16	2.91	0.61	2.65	0.10	5.58	0.17	4.58	0.33
CTBP2	3.35	0.19	2.98	0.37	1.28	0.75	2.59	0.37	4.82	0.15	7.92	1.05
CTCF	4.89	0.22	4.75	0.38	4.02	0.53	4.64	0.48	7.10	0.05	8.71	4.95
DNMT1	4.68	0.54	4.09	0.26	4.24	0.66	2.07	2.49	6.87	0.09	6.31	0.40
DNMT3A	5.11	0.21	4.79	0.08	3.55	0.64	6.67	0.63	7.73	0.13	9.18	0.50
DNMT3B	2.63	0.12	7.74	0.44	6.79	0.63	10.84	0.86	9.35	0.27	11.24	0.47
DOT1L	7.18	0.03	5.78	1.23	5.63	0.85	7.90	0.96	10.06	0.16	9.30	0.27
DZIP3	6.10	0.19	5.75	0.66	4.27	0.81	4.23	0.57	7.27	0.12	7.35	0.12
EED	6.03	0.10	5.12	0.39	5.29	0.58	4.44	0.33	6.74	0.08	7.83	2.64
EHMT2	4.74	0.10	4.51	0.34	3.90	0.48	4.25	0.30	8.49	0.17	8.17	0.53
ESCO1	6.32	0.26	6.20	0.83	5.57	0.79	5.19	0.41	6.29	0.09	5.48	0.11
ESCO2	7.32	0.13	5.73	0.28	11.30	0.21	11.15	0.25	10.37	0.26	10.42	0.69
EZH1	8.92	0.81	7.07	0.75	5.83	0.55	-2.75	0.59	8.63	0.12	7.65	0.46
EZH2	6.01	0.53	3.91	0.13	4.19	0.57	8.61	0.63	8.26	0.18	9.30	2.52
HAT1	5.91	0.16	4.70	0.13	6.32	0.04	5.06	0.25	6.56	0.11	5.55	0.14
HDAC1	4.81	0.25	4.40	0.33	5.96	0.38	3.90	0.59	5.43	0.12	4.54	0.42
HDAC10	8.84	0.40	8.73	1.09	6.79	0.60	7.12	0.29	10.73	0.49	8.38	0.12
HDAC11	9.12	0.38	10.56	1.20	7.95	0.81	6.28	0.78	10.64	0.19	10.41	0.98
HDAC2	12.68	0.66	11.38	0.87	8.03	0.23	9.26	0.19	11.13	0.08	11.06	1.30
HDAC3	5.84	0.14	5.37	0.41	4.67	0.47	4.78	0.33	5.29	0.05	4.60	0.29
HDAC4	6.56	0.17	5.40	1.06	5.11	0.51	3.61	0.47	8.44	0.23	8.04	0.43
HDAC5	10.76	0.15	9.29	1.01	6.32	1.15	6.16	0.40	12.81	0.11	13.02	1.43
HDAC6	7.61	0.15	6.64	0.43	4.02	0.66	4.71	0.35	7.29	0.11	4.34	0.53
HDAC7	5.01	1.09	5.67	1.14	3.83	1.08	1.28	0.95	7.56	0.14	7.80	0.29
HDAC8	8.41	0.33	6.37	0.58	5.21	0.56	5.64	0.52	8.22	0.02	6.49	0.47
HDAC9	13.61	0.13	13.51	1.79	10.01	1.01	7.92	0.82	12.44	0.30	13.16	2.18
ING1	6.74	0.49	6.54	0.23	5.79	0.60	5.22	0.21	7.46	0.10	6.41	1.42
ING2	6.60	0.20	6.19	0.08	5.87	1.06	5.25	0.19	7.02	0.18	6.69	1.18
ING3	7.92	0.32	6.46	0.07	5.61	0.33	6.05	0.26	7.56	0.07	6.41	0.72
ING4	8.11	0.29	6.82	0.32	4.52	0.42	6.22	0.10	8.45	0.34	7.24	0.70
ING5	7.63	0.23	7.04	0.52	7.05	0.72	6.20	0.14	9.43	0.24	8.24	0.30
INO80	8.64	0.19	7.06	0.45	7.23	0.90	7.25	0.20	10.00	0.35	8.38	0.36
KAT2A	6.09	0.08	5.22	0.75	6.09	0.55	4.06	0.62	8.00	0.19	6.15	0.76
KAT2B	12.08	0.06	9.43	0.65	6.76	0.24	3.88	0.43	8.42	0.34	4.56	0.15
KAT5	8.74	0.03	7.18	0.23	7.06	0.37	6.89	0.60	9.64	0.21	8.14	0.38
KDM1	4.03	0.09	2.46	0.21	2.02	0.33	4.33	0.40	5.82	0.06	6.38	0.40
KDM4A	5.31	0.32	5.56	0.43	4.11	0.38	5.35	0.23	8.00	0.22	6.41	0.44
KDM4C	9.66	0.21	8.79	1.04	8.66	0.68	8.23	0.17	9.81	0.02	8.00	0.19
KDM5B	4.54	0.15	6.21	0.19	3.12	0.36	5.73	0.82	5.24	0.22	7.34	0.70
KDM5C	10.72	0.61	10.67	0.19	9.27	0.44	11.63	1.37	11.28	0.17	12.18	0.39
KDM6B	7.47	0.12	6.35	0.27	4.01	0.44	7.10	0.07	8.02	0.14	7.75	0.52
MBD1	7.90	0.12	7.03	0.47	6.05	0.79	6.49	0.18	9.14	0.25	8.93	0.95
MBD2	5.47	0.08	4.88	0.26	3.52	0.57	3.21	0.27	5.03	0.12	3.69	0.34
MBD3	6.67	0.26	6.12	0.32	5.47	0.57	5.05	0.31	7.99	0.35	6.88	0.70

gene	ΔCt H9	SD	ΔCt Lu d0	SD	ΔCt Lu d6	SD	ΔCt Ctx	SD	ΔCt Hep-like	SD	ΔCt huHep	SD
MBD4	5.48	0.11	5.10	0.45	4.78	0.76	3.80	0.23	5.62	0.09	4.19	0.41
MECP2	9.31	0.08	7.55	0.64	4.86	0.53	4.51	0.16	9.02	0.10	9.29	0.36
MLL	6.80	0.32	5.54	0.90	3.80	1.08	4.65	0.65	8.02	0.12	6.54	0.37
MLL3	8.04	0.14	7.57	0.56	4.76	0.94	5.89	0.63	8.82	0.03	6.82	0.20
MLL5	6.87	0.16	6.24	0.73	3.52	0.65	3.29	0.39	6.79	0.16	5.55	0.03
MTA1	3.88	0.30	3.02	0.28	3.18	0.71	4.14	0.18	6.33	0.37	6.38	0.49
MTA2	6.50	0.42	5.35	0.13	4.33	0.53	6.10	0.70	9.23	0.22	8.18	0.06
MYSM1	5.30	0.26	5.83	1.03	4.76	0.89	4.28	0.33	6.60	0.22	5.50	0.09
MYST1	6.05	0.22	4.92	0.20	5.12	0.45	4.21	0.34	7.11	0.04	6.15	0.13
MYST2	4.97	0.57	5.49	0.20	5.05	0.28	5.05	0.52	7.05	0.16	6.50	0.28
MYST3	6.30	0.41	4.35	0.56	2.99	0.83	2.27	0.74	6.48	0.25	4.76	0.38
MYST4	15.75	0.14	12.93	2.69	10.16	1.97	8.84	0.63	15.56	1.05	13.81	0.89
NCOA1	14.53	0.71	13.17	0.94	9.09	0.50	9.34	1.05	10.94	0.20	10.46	0.31
NCOA3	7.31	0.21	7.62	0.77	5.99	0.37	5.64	0.26	7.37	0.16	6.13	0.23
NEK6	7.10	0.20	4.60	0.34	6.48	0.70	3.50	0.65	5.98	0.11	4.18	0.59
NSD1	5.19	0.16	5.22	0.64	4.55	0.74	3.61	0.25	7.41	0.19	6.59	0.44
PAK1	4.42	0.30	5.82	0.14	4.04	0.45	4.59	0.91	6.12	0.13	6.44	0.20
PBRM1	5.76	0.18	3.64	0.54	1.84	0.89	3.65	0.20	5.93	0.11	4.00	0.15
PCGF1	7.81	0.03	5.89	0.22	5.76	0.52	5.96	0.21	7.75	0.18	6.75	0.33
PCGF2	5.49	0.22	5.07	0.33	3.74	0.71	5.03	0.22	6.46	0.22	8.08	0.32
PCGF5	8.46	0.31	7.52	0.38	11.09	0.28	3.05	0.31	6.27	0.13	3.22	0.34
PHC1	2.37	0.35	5.71	0.22	4.17	0.59	5.28	0.62	7.80	0.22	8.90	0.38
PHC2	11.88	0.51	7.44	0.56	5.71	0.33	8.15	0.31	8.14	0.34	7.23	0.90
PHF1	6.31	1.15	5.65	0.82	3.77	0.91	2.42	0.44	5.13	0.06	4.88	0.32
PHF13	6.35	0.43	6.44	0.22	5.67	1.01	7.04	0.21	8.12	0.21	7.52	0.29
PHF2	8.67	0.38	6.84	0.20	5.27	0.24	4.82	0.39	7.18	0.30	5.91	0.34
PHF21A	7.16	0.53	5.70	0.50	4.38	0.92	4.70	0.41	7.84	0.18	8.07	0.14
PRMT1	2.74	0.25	1.75	0.33	1.92	0.31	3.51	0.19	4.88	0.03	5.49	0.19
PRMT2	5.99	0.10	5.88	0.43	3.27	0.71	3.49	0.37	6.04	0.12	6.46	0.25
PRMT3	6.59	0.44	5.18	0.31	6.70	0.22	5.76	0.82	7.74	0.12	6.66	0.24
PRMT5	6.15	0.32	5.11	0.15	5.88	0.03	6.16	0.68	6.43	0.08	6.37	0.08
PRMT6	10.76	0.19	10.65	0.36	9.80	0.21	10.42	1.16	13.31	0.67	11.79	0.49
PRMT7	7.78	0.04	6.25	0.90	5.76	0.84	5.39	0.45	7.40	0.02	6.97	0.13
PRMT8	10.71	0.15	15.50	1.13	8.22	1.64	6.22	1.22	17.29	1.96	14.18	1.70
RING1	6.10	0.33	5.35	0.22	3.48	0.57	2.79	0.44	5.56	0.09	3.60	0.70
RNF2	5.82	0.25	5.37	0.27	4.26	0.32	5.81	0.38	5.54	0.10	6.00	0.58
RNF20	6.77	0.19	6.20	0.23	4.95	0.04	4.72	0.50	6.66	0.24	5.37	0.18
RPS6KA3	8.54	0.52	7.33	0.36	7.64	0.60	4.23	0.35	5.66	0.11	4.19	0.26
RPS6KA5	9.67	0.29	8.14	0.85	7.73	1.26	4.88	0.40	9.32	0.10	8.40	0.52
SETD1A	6.27	0.08	5.39	0.33	5.08	0.39	5.58	0.37	9.21	0.21	7.55	0.33
SETD1B	8.42	0.24	7.73	0.71	5.76	0.66	6.74	0.30	9.87	0.18	7.16	0.66
SETD2	5.88	0.20	4.94	0.54	3.65	0.59	3.33	0.60	6.74	0.10	4.77	0.15
SETD3	5.92	0.07	5.78	0.41	4.88	0.36	3.55	0.55	5.50	0.06	4.70	0.07
SETD6	8.20	0.16	6.39	0.72	6.33	0.65	6.96	0.42	9.16	0.22	6.51	0.24
SETD7	10.06	0.39	5.74	0.42	5.02	0.52	4.15	0.33	6.98	0.13	3.82	0.12
SETD8	8.12	0.10	5.73	0.38	6.42	0.49	5.74	0.42	8.08	0.10	7.03	0.28
SETDB1	7.82	0.09	6.42	0.27	5.66	0.42	7.24	0.42	8.24	0.11	6.80	0.18
SETDB2	7.91	0.22	6.91	0.33	5.91	0.30	5.08	0.29	7.79	0.12	5.51	0.29

SMARCA2	9.22	0.27	8.53	0.41	7.82	1.22	2.94	0.28	6.55	0.03	3.59	0.71
SMARCA4	4.46	0.65	3.10	0.46	1.91	0.99	4.02	0.15	6.39	0.28	6.35	0.34
SMYD3	6.79	0.43	4.88	0.27	3.91	0.41	5.68	0.81	5.19	0.22	7.59	1.25
SPEN	6.43	0.40	5.10	0.64	4.04	0.92	4.00	0.46	7.57	0.28	6.37	2.25
SUV39H1	8.91	0.08	7.64	0.39	8.32	1.04	7.81	0.46	10.60	0.39	9.33	0.38
SUV420H1	5.15	0.05	5.36	0.17	3.04	0.50	3.19	0.36	5.67	0.15	4.59	0.07
SUZ12	6.03	0.23	5.10	0.11	5.10	0.26	5.48	0.04	5.57	0.02	4.66	0.64
TET1	6.09	0.91	5.66	0.76	4.72	1.15	5.93	0.63	6.93	0.41	11.85	1.67
TET2	11.33	0.68	7.57	0.52	5.00	0.11	4.66	0.08	8.33	0.25	6.57	0.09
UBE2A	6.25	0.55	6.68	0.60	4.30	0.59	3.08	0.47	5.51	0.18	4.97	0.42
UBE2B	5.48	0.38	4.23	0.13	3.26	0.26	2.55	0.26	4.76	0.04	2.82	0.07
USP16	7.83	0.20	6.05	0.50	6.12	0.55	4.42	0.54	6.95	0.02	5.00	0.22
USP21	7.58	0.05	6.46	0.61	5.79	0.94	4.55	0.48	8.25	0.05	8.47	0.17
USP22	4.37	0.18	3.80	0.33	2.25	0.66	3.76	0.33	6.51	0.21	6.81	0.77
WHSC1	6.08	0.09	4.11	0.33	3.62	0.60	4.70	0.54	7.91	0.14	7.34	0.14
