# Supplementary material for: Lineage-Specific Regulation of Epigenetic Modifier Genes in Human Liver and Brain
Source: PLoS One. 2014 Jul 23;9(7):e102035. doi: 10.1371/journal.pone.0102035 (PMC4108363; doi:10.1371/journal.pone.0102035)
Supplement: Figure S4 — Expression of epigenetic regulators genes in LU d6 relative to d0. (A) RNA was prepared from proliferating LUHMES cells (d0), or the same cells after six days of neuronal differentiation (d6). EMG expression was determined by means of RT-qPCR. The threshold cycle values were determinded with the CFX96 optical system software. Relative gene expression values were calculated by normalization to house keeping genes. Lu d6 data were normalized to d0 expressions (the latter set to 1). Data are means of three independent differentiation. (B) p-values were calculated with the SAB online analysis tool and correspond to the statistical difference from the expression levels in Lu d0. SEM were calculated by GraphPad Prism Software. (PDF) [file pone.0102035.s004.pdf]

Figure S4: Expression of epigenetic regulators genes in LU d6 relative to d0

A LUHMES d6 expression levels (relative to d0)

Gene	neurons
AURKB	
ESCO2	
PCGF5	
AURKA	N
NEK6	N
HAT1	N
HDAC1	N
PRMT3	N
BAF45A	N
KAT2A	
PRMT5	
PCGF6	
SETD8	
SUV39H1	
BAF53A	
CDYL	
BRD7	
EZH2	
RPS6KA3	
EED	
INO80	
PRMT1	
MYST1	
BAZ1A	
DNMT1	
ING5	
SUZ12	
USP16	
CHD1	
PCGF1	
SETD6	
PHF5A	
KAT5	
BAF60C	
DOT1L	
CBX3	
KDM4C	
ING2	
MBD4	
SETD1A	
BAF60A	
HDAC4	
CBX6	
CBX5	
BAZ1B	
MYST2	
KDM1	
RPS6KA5	
PRMT7	
TET1	
PHF6	
CARM1	
PHF3	
CTCF	
WHSC1	
MBD3	
ESCO1	
BRD2	
USP21	
ING1	
PHF13	
EHMT2	
NSD1	
SETDB1	
SETD7	
NAB2	
BRD8	
HDAC3	
SETD4	
SMARCA2	
EZH1	
CSRP2BP	
BRD4	
ING3	
BRDT	
CHD7	
CBX1	
CTBP1	
BRWD3	
PRMT6	
SETD3	
CHD2	
CHD5	

Gene	neurons
MBD1	
DNMT3B	
SPEN	
SETDB2	
UBE2B	
MYSM1	
SMYD3	
BAZ2A	
HDAC8	
RNF2	
SMARCA4	
ASXL1	
MBD2	
PHF21A	
AURKC	
RNF20	
BRWD1	
PCGF2	
MBD2	
DNMT3A	
SETD5	
CHD4	
SETD2	
CHD8	
KDM5C	
NCOA6	
BMI1	
PHF2	
ARID1A	
MYST3	
PHC1	
KDM4A	
DZIP3	
ASH1L	
CHD6	
USP22	
BPTF	
CTBP2	
CBX7	
PHC2	
CBX8	
PHF7	
NCOA3	
PBRM1	
PCGF3	
RING1	
PAK1	
MLL	
PHF1	
CHD9	
HDAC7	
ATF2	
BRD3	
BRPF1	
BRPF3	
BRD1	
HDAC10	
SETD1B	
CBX4	
TET2	
CHD3	
CIITA	
ING4	
UBE2A	
KDM6B	
SUV420H1	
MECP2	
HDAC11	
HDAC6	
KAT2B	
PRMT2	
MLL5	
MYST4	
MLL3	
HDAC5	
HDAC9	
KDM5B	
BAZ2B	
HDAC2	
PHF21B	
NCOA1	
CDYL2	
PRMT8	
BAF53B	

B Complete data set on LUHMES d6 versus d0 comparison

Gene	rel. Expression	SEM	p-value
ARID1A	4.6	1.43	0.074
ASH1L	4.4	1.21	0.049
ASXL1	4.5	2.35	0.228
ATF2	5.6	1.39	0.032
AURKA	0.4	0.15	0.026
AURKB	0.0	0.01	0.007
AURKC	4.0	1.66	0.186
BAF45A	1.2	0.89	0.821
BAF53A	1.1	0.85	0.399
BAF53B	6037.7	4482.34	0.039
BAF60A	4.7	4.09	0.176
BAF60C	2.2	1.60	0.275
BAZ1A	1.4	0.27	0.327
BAZ1B	2.0	0.37	0.055
BAZ2A	3.3	0.69	0.030
BAZ2B	15.5	7.17	0.113
BMI1	4.7	1.76	0.103
BPTF	4.8	1.11	0.027
BRD1	5.8	0.69	0.002
BRD2	2.3	0.38	0.029
BRD3	5.6	1.02	0.011
BRD4	2.8	0.91	0.131
BRD7	1.1	0.18	0.524
BRD8	2.7	0.90	0.144
BRDT	2.7	0.75	0.092
BRPF1	5.5	0.36	0.000
BRPF3	6.6	2.32	0.073
BRWD1	4.5	2.17	0.191
BRWD2	2.6	0.83	0.139
BRWD3	2.9	0.95	0.121
CARM1	2.0	0.20	0.011
CBX1	2.7	0.58	0.043
CBX3	1.6	0.18	0.051
CBX4	6.7	1.46	0.018
CBX5	2.0	0.43	0.080
CBX6	1.9	0.34	0.078
CBX7	5.3	2.11	0.117
CBX8	4.8	0.88	0.013
CDYL	1.1	0.02	0.723
CDYL2	48.8	21.81	0.094
CHD1	1.6	0.42	0.260
ING5	1.5	0.42	0.380
INO80	1.4	0.45	0.482
KAT2A	0.8	0.21	0.526
KAT2B	9.3	0.88	0.001
KAT5	1.6	0.22	0.078
KDM1	2.0	0.27	0.028

Gene	rel. Expression	SEM	p-value
CHD2	3.4	1.64	0.234
CHD3	7.7	3.38	0.118
CHD4	4.5	1.77	0.123
CHD5	4.4	2.91	0.314
CHD6	4.3	0.45	0.002
CHD7	2.8	0.82	0.115
CHD8	4.6	1.81	0.122
CHD9	5.6	1.27	0.023
CIITA	10.5	6.81	0.244
CSRP2BP	2.4	0.21	0.003
CTBP1	2.7	0.59	0.046
CTBP2	5.0	1.62	0.070
CTCF	2.3	0.44	0.056
DNMT1	1.4	0.46	0.408
DNMT3A	3.8	0.84	0.031
DNMT3B	3.0	0.82	0.075
DOT1L	1.8	0.65	0.528
DZIP3	4.6	1.63	0.100
EED	1.3	0.28	0.512
EHMT2	2.4	0.49	0.053
ESCO1	2.4	0.84	0.226
ESCO2	0.0	0.00	0.000
EZH1	3.2	2.34	0.064
EZH2	1.2	0.24	0.484
HAT1	0.5	0.01	0.000
HDAC1	0.5	0.08	0.021
HDAC10	6.0	1.60	0.043
HDAC11	9.7	2.98	0.052
HDAC2	17.7	2.76	0.004
HDAC3	2.5	0.51	0.050
HDAC4	1.9	0.44	0.345
HDAC5	14.0	6.88	0.137
HDAC6	9.5	2.46	0.026
HDAC7	6.4	3.02	0.176
HDAC8	3.3	0.89	0.067
HDAC9	13.2	4.14	0.045
HINFP	3.2	0.58	0.020
ING1	2.5	0.58	0.068
ING2	2.1	0.80	0.248
ING3	2.5	0.26	0.004
ING4	7.4	1.26	0.007
PRMT2	10.1	2.47	0.022
PRMT3	0.5	0.06	0.021
PRMT5	0.8	0.02	0.067
PRMT6	2.6	0.17	0.001
PRMT7	2.2	0.81	0.292
PRMT8	159.7	76.79	0.108

Gene	rel. Expression	SEM	p-value
KDM4A	4.1	0.61	0.009
KDM4C	1.7	0.46	0.401
KDM5B	12.2	1.52	0.002
KDM5C	4.1	0.74	0.015
KDM6B	7.5	1.28	0.007
MBD1	3.1	0.89	0.086
MBD2	3.5	0.59	0.013
MBD3	2.3	0.46	0.053
MBD4	1.9	0.55	0.199
MECP2	9.2	1.99	0.015
MLL	5.8	2.22	0.102
MLL3	11.7	3.69	0.045
MLL5	10.0	2.22	0.016
MTA1	1.4	0.37	0.436
MTA2	2.9	0.63	0.041
MYSM1	3.5	1.32	0.167
MYST1	1.3	0.29	0.342
MYST2	1.9	0.20	0.011
MYST3	4.4	1.33	0.071
MYST4	19.1	14.53	0.333
NAB2	2.8	0.93	0.129
NCOA1	25.5	4.67	0.007
NCOA3	4.7	0.79	0.013
NCOA6	4.2	1.08	0.043
NEK6	0.4	0.11	0.018
NSD1	2.5	0.77	0.141
PAK1	5.0	0.72	0.005
PBRM1	5.6	2.02	0.091
PCGF1	1.6	0.34	0.185
PCGF2	3.8	1.16	0.072
PCGF3	5.4	1.74	0.081
PCGF5	0.1	0.01	0.004
PCGF6	1.1	0.44	0.931
PHC1	4.3	0.85	0.019
PHC2	4.7	0.55	0.005
PHF1	6.0	2.35	0.111
PHF13	2.7	0.96	0.151
PHF2	4.1	0.40	0.002
PHF21A	4.0	1.57	0.138
PHF21B	20.8	5.22	0.019
PHF3	2.2	0.42	0.063
PHF5A	1.6	0.37	0.169
PHF6	2.1	0.35	0.037
PHF7	4.9	1.01	0.019
PRMT1	1.3	0.12	0.242

Gene	rel. Expression	SEM	p-value
RING1	5.2	1.34	0.035
RNF2	3.2	0.11	0.000
RNF20	3.4	0.12	0.000
RPS6KA3	1.2	0.31	0.510
RPS6KA5	2.4	1.07	0.288
SETD1A	1.8	0.25	0.045
SETD1B	6.1	1.62	0.037
SETD2	3.9	0.81	0.027
SETD3	2.7	0.40	0.017
SETD4	2.5	0.53	0.083
SETD5	4.1	1.34	0.088
SETD6	1.5	0.36	0.366
SETD7	2.5	0.49	0.046
SETD8	0.9	0.17	0.622
SETDB1	2.4	0.40	0.026
SETDB2	2.9	0.30	0.004
SMARCA2	3.0	1.16	0.164
SMARCA4	3.8	1.41	0.125
SMYD3	3.1	0.45	0.010
SPEN	3.4	1.32	0.165
SUV39H1	1.1	0.49	0.875
SUV420H1	7.9	1.40	0.008
SUZ12	1.4	0.11	0.026
TET1	3.2	2.23	0.237
TET2	9.5	7.87	0.000
TRIM27	2.4	0.40	0.027
UBE2A	7.6	1.79	0.022
UBE2B	3.0	0.24	0.001
USP16	1.4	0.34	0.363
USP21	2.5	0.97	0.209
USP22	4.5	1.01	0.026
WHSC1	2.2	0.46	0.061
ZMYND8	3.4	1.18	0.122
