# Supplementary material for: Lineage-Specific Regulation of Epigenetic Modifier Genes in Human Liver and Brain
Source: PLoS One. 2014 Jul 23;9(7):e102035. doi: 10.1371/journal.pone.0102035 (PMC4108363; doi:10.1371/journal.pone.0102035)
Supplement: Figure S5 — Comparison of Ctx and Lu6 gene expression with mesencephalic tissue gene array data and iPSC derived dopaminergic neurons. Data from a study on dopaminergic neurons (DA d42) that were derived from induced pluripotent stem cells (iPSC) and fetal mesencephalon tissue (huMES) were retrieved from a data base and compared to Lu d6 and Ctx [31]. Microarray data (GSE51214) from this work were normalized to pluripotent stem cells. Data for the 156 EMGs were obstained from this data set. All expression values are given as log2 fold-change values compared to the prespective reference cell source (pluripotent stem cells). A heat map was generated for visualization. The heat map depicts every log fold change <1 with N for “not regulated”, as we defined a cutoff of 2-fold expression changes (1 on the log2 scale). (PDF) [file pone.0102035.s005.pdf]

Figure S5: Comparison of Ctx and Lu6 gene expression with mesencephalic tissue gene array data and iPSC derived dopaminergic neurons

Symbol	Lu6	Ctx	DA d42	huMES	Lu d6	CTX	DA d42	huMES
AURKB	-4.15	-4.12	-2.83	-1.76				
DNMT3B	-4.07	-8.07	-4.61	-3.80				
ESCO2	-3.97	-3.82	-1.90	-0.89				N
PCGF5	-2.61	5.44	0.76	0.68			N	N
HDAC1	-1.12	0.99	-2.18	-3.11		N		
AURKA	-0.99	-0.78	-3.15	-2.41	N	N		
BRDT	-0.85	1.12	-0.05	0.12	N		N	N
CDYL	-0.81	-0.48	-1.22	-0.55	N	N		N
HAT1	-0.41	0.87	-1.38	-1.02	N	N		
PRMT3	-0.10	0.97	-1.39	-1.37	N	N		
MYST2	-0.06	-0.02	-0.89	-0.56	N	N	N	N
KAT2A	0.07	2.11	0.15	-0.87	N		N	N
PRMT5	0.26	0.10	-0.67	-0.69	N	N	N	N
AURKC	0.41	0.58	-1.00	-0.81	N	N	N	N
PAK1	0.42	0.04	-1.09	-0.83	N	N		N
DNMT1	0.55	3.51	-1.01	-0.67	N			N
BRD7	0.68	1.29	-1.17	-1.39	N			
ING5	0.70	1.43	-0.66	-0.71	N		N	N
MYSM1	0.73	1.05	-0.82	-1.43	N		N	
NEK6	0.73	3.69	0.04	-0.36	N		N	N
NSD1	0.77	1.59	-1.15	-0.60	N			N
MTA1	0.81	-0.25	-0.48	-0.11	N	N	N	N
EED	0.82	1.62	-0.52	-0.24	N		N	N
MBD4	0.83	1.70	-0.88	-1.16	N		N	
SUV39H1	0.86	1.15	-1.24	-0.85	N			N
EHMT2	0.89	0.50	-0.73	-0.60	N	N	N	N
PHF13	0.90	-0.68	-0.80	-0.55	N	N	N	N
ESCO1	0.90	1.16	-1.14	-1.46	N			
BAF45A	0.91	4.58	-0.08	-0.21	N		N	N
CTCF	0.93	0.31	-0.98	-0.20	N	N	N	N
SUZ12	0.95	0.55	-0.41	-0.13	N	N	N	N
PRMT6	0.97	0.62	-0.55	0.04	N	N	N	N
ING2	0.97	1.36	0.10	-0.07	N		N	N
RPS6KA3	0.97	4.33	1.03	-0.57	N			N
MYST1	0.98	1.87	-0.26	0.05	N		N	N
ING1	1.03	1.53	-0.16	0.00			N	N
CARM1	1.03	-0.82	-0.74	-0.46		N	N	N
SETD3	1.07	2.43	-0.49	-0.68			N	N
CBX7	1.10	6.27	-0.30	-0.56			N	N
KDM4C	1.11	1.43	-0.40	-1.07			N	
SETD1A	1.22	0.71	0.18	0.54		N	N	N
HDAC3	1.23	1.08	-0.74	-0.87			N	N
KDM4A	1.23	-0.03	-1.27	-0.79		N		N
MBD3	1.27	1.64	0.38	0.78			N	N

Symbol	Lu6	Ctx	DA d42	huMES	Lu d6	CTX	DA d42	huMES
BAF53A	1.29	5.11	-1.76	-0.84				N
CHD5	1.30	6.39	2.08	1.08				
HDAC11	1.31	2.96	0.74	-0.14			N	N
NCOA3	1.36	1.69	-0.36	-0.27			N	N
CBX1	1.38	-0.02	-0.53	-1.28		N	N	
KDM5B	1.44	-1.03	-0.67	-0.33			N	N
HDAC7	1.46	3.90	-0.45	-0.13			N	N
CBX3	1.48	-0.43	-0.20	0.11		N	N	N
KDM5C	1.49	-0.54	-0.34	-0.82		N	N	N
HDAC4	1.50	3.00	0.30	0.63			N	N
RNF2	1.58	0.04	-0.29	0.63		N	N	N
INO80	1.59	1.39	-0.51	-0.69			N	N
DNMT3A	1.65	-1.48	-0.40	-0.24			N	N
TET1	1.68	0.25	-1.51	-0.28		N		N
SMARCA2	1.68	6.29	0.77	-0.06			N	N
BRPF3	1.70	0.98	0.23	0.27		N	N	N
BAF60A	1.70	2.23	-0.85	-0.42			N	N
KAT5	1.71	1.92	-0.01	0.14			N	N
CBX5	1.71	0.25	-0.76	-0.80		N	N	N
BAZ1B	1.71	0.62	-0.18	0.14		N	N	N
DOT1L	1.72	-0.54	-0.11	-0.09		N	N	N
SETD8	1.75	2.42	0.28	0.55			N	N
USP16	1.78	3.47	-0.03	-0.49			N	N
MBD2	1.81	2.38	0.25	0.27			N	N
RNF20	1.83	2.11	-0.56	-0.11			N	N
BRD4	1.84	0.21	0.16	0.81		N	N	N
PCGF2	1.87	0.47	0.28	0.72		N	N	N
CHD1	1.88	1.35	-1.25	-1.18				
BRD3	1.88	-1.24	-0.16	0.17			N	N
EZH2	1.89	-2.51	-1.18	-0.40				N
CTBP1	1.93	2.11	0.38	0.43			N	N
BRD8	1.96	2.10	2.92	2.19				
SETD6	1.96	1.28	-0.55	-0.66			N	N
DZIP3	1.99	1.95	0.12	-0.84			N	N
MBD1	1.99	1.42	0.17	-0.42			N	N
USP21	2.00	3.08	-0.04	-0.10			N	N
CHD8	2.00	1.40	-0.48	-0.42			N	N
BAZ2A	2.02	0.71	-0.72	-0.49		N	N	N
SETDB2	2.02	2.84	-0.07	0.23			N	N
KDM1	2.03	-0.27	-0.91	-0.50		N	N	N
UBE2A	2.03	3.22	0.19	-0.83			N	N
CDYL2	2.11	4.56	0.97	0.32			N	N
PCGF1	2.12	1.87	-0.26	-0.28			N	N
HDAC10	2.14	1.74	0.37	-0.19			N	N
SUV420H1	2.17	1.99	-0.06	-0.31			N	N
PRMT7	2.19	2.43	0.00	-0.35			N	N
BPTF	2.19	2.43	-0.19	0.21			N	N
SETDB1	2.19	0.62	-0.67	-0.15		N	N	N
CTBP2	2.20	0.79	-0.18	-0.01		N	N	N

Symbol	Lu6	Ctx	DA d42	huMES	Lu d6	CTX	DA d42	huMES
USP22	2.21	0.63	0.43	0.61		N	N	N
UBE2B	2.23	2.95	0.36	0.70			N	N
MTA2	2.24	0.53	-1.00	-0.31		N	N	N
RPS6KA5	2.27	4.83	-0.34	0.50			N	N
BRPF1	2.29	1.56	-0.40	-0.24			N	N
SETD2	2.31	2.64	0.54	0.74			N	N
BRWD1	2.32	2.74	-0.94	-0.66			N	N
ING3	2.33	1.89	-0.14	-0.23			N	N
CHD2	2.36	2.21	0.10	-0.11			N	N
CHD6	2.39	2.13	-0.58	-0.33			N	N
WHSC1	2.54	1.45	-0.07	0.12			N	N
ARID1A	2.54	0.74	0.10	0.97		N	N	N
SPEN	2.59	2.48	0.31	0.75			N	N
CBX6	2.68	3.57	1.32	0.57				N
RING1	2.70	3.36	1.02	0.72				N
PHF1	2.74	3.94	0.66	-0.45			N	N
SMARCA4	2.76	0.44	0.01	0.28		N	N	N
SETD1B	2.76	1.70	0.15	1.09			N	
PRMT2	2.83	2.53	1.11	0.78				N
ATF2	2.90	3.53	1.13	1.03				
SMYD3	2.92	1.27	-0.23	-0.72			N	N
PHF21A	2.99	2.50	0.75	0.65			N	N
PRMT8	2.99	4.83	0.79	0.60			N	N
CHD7	3.03	1.67	-0.80	-0.49			N	N
CHD4	3.04	1.19	-0.66	-0.48			N	N
BRD1	3.12	0.90	0.13	0.69		N	N	N
EZH1	3.16	11.75	0.46	-0.13			N	N
MLL	3.25	2.24	-0.27	0.37			N	N
HDAC8	3.27	2.83	0.27	-1.55			N	
PHF2	3.41	3.88	1.73	1.68				
MLL5	3.43	3.61	0.89	0.97			N	N
MLL3	3.46	2.23	0.62	0.77			N	N
MYST3	3.46	4.17	0.23	0.26			N	N
KDM6B	3.50	0.37	1.21	1.99		N		
CBX4	3.51	2.60	1.32	1.17				
ING4	3.63	1.90	0.51	0.63			N	N
HDAC6	3.68	2.93	0.93	0.88			N	N
CHD9	3.78	3.18	0.33	0.68			N	N
HDAC9	3.80	5.86	0.87	0.91			N	N
CHD3	3.86	2.12	1.40	1.88				
PBRM1	4.10	2.12	0.21	0.77			N	N
BAZ2B	4.36	3.78	0.65	0.89			N	N
MECP2	4.51	4.81	0.59	1.22			N	
BAZ1A	4.53	2.88	-0.90	0.25			N	N
HDAC2	4.66	3.42	-0.51	-0.20			N	N
BAF60C	4.71	9.83	1.92	2.19				
ASXL1	4.74	4.90	1.21	1.53				
HDAC5	4.76	4.64	0.27	0.10			N	N
SETD7	5.10	5.94	1.79	0.69				N

Symbol	Lu6	Ctx	DA d42	huMES
CBX8	5.28	3.22	0.57	0.40
KAT2B	5.34	8.25	2.45	2.52
BMI1	5.48	5.11	1.73	1.95
NCOA1	5.49	5.45	0.95	1.24
PHC2	6.20	3.75	1.21	1.97
TET2	6.33	6.67	2.58	2.93
MYST4	6.53	7.01	0.29	1.18
BAF53B	12.75	10.12	3.54	3.35

Lu d6	CTX	DA d42	huMES
		N	N
		N	
		N	
